# Complementary Analytical Platforms of NMR Spectroscopy and LCMS Analysis in the Metabolite Profiling of *Isochrysis galbana*

**DOI:** 10.3390/md19030139

**Published:** 2021-03-02

**Authors:** Muhammad Safwan Ahamad Bustamam, Hamza Ahmed Pantami, Awanis Azizan, Khozirah Shaari, Chong Chou Min, Faridah Abas, Norio Nagao, Maulidiani Maulidiani, Sanjoy Banerjee, Fadzil Sulaiman, Intan Safinar Ismail

**Affiliations:** 1 Natural Medicine and Products Research Laboratory, Institute of Bioscience, Universiti Putra Malaysia, Serdang 43400, Selangor, Malaysia; safwan.upm@gmail.com (M.S.A.B.); awanis_azizan@yahoo.com (A.A.); khozirah@upm.edu.my (K.S.); faridah_abas@upm.edu.my (F.A.); sanjoybanerjee@gmail.com (S.B.); mfadzilsulaiman90@gmail.com (F.S.); 2 Department of Chemistry, Faculty of Science, Universiti Putra Malaysia, Serdang 43400, Selangor, Malaysia; hamza3983@gmail.com; 3 Department of Aquaculture, Faculty of Agriculture, Universiti Putra Malaysia, Serdang 43400, Selangor, Malaysia; choumin@upm.edu.my (C.C.M.); norionagao@gmail.com (N.N.); 4 Faculty of Science and Marine Environment, Universiti Malaysia Terengganu, Kuala Nerus 21030, Terengganu, Malaysia; maulidiani@umt.edu.my

**Keywords:** *Isochrysis galbana*, metabolite profile, metabolomics, NMR, LCMS, molecular network

## Abstract

This study was designed to profile the metabolites of *Isochrysis galbana*, an indigenous and less explored microalgae species. ^1^H Nuclear Magnetic Resonance (NMR) spectroscopy and Liquid Chromatography-Mass Spectrometry (LCMS) were used to establish the metabolite profiles of five different extracts of this microalga, which are hexane (Hex), ethyl acetate (EtOAc), absolute ethanol (EtOH), EtOH:water 1:1 (AqE), and 100% water (Aq). Partial least square discriminant analysis (PLS–DA) of the generated profiles revealed that EtOAc and Aq extracts contain a diverse range of metabolites as compared to the other extracts with a total of twenty-one metabolites, comprising carotenoids, polyunsaturated fatty acids, and amino acids, that were putatively identified from the NMR spectra. Meanwhile, thirty-two metabolites were successfully annotated from the LCMS/MS data, ten of which (palmitic acid, oleic acid, α-linolenic acid, arachidic acid, cholesterol, DHA, DPA, fucoxanthin, astaxanthin, and pheophytin) were similar to those present in the NMR profile. Another eleven glycerophospholipids were discovered using MS/MS-based molecular network (MN) platform. The results of this study, besides providing a better understanding of *I.* *galbana*’s chemical make-up, will be of importance in exploring this species potential as a feed ingredient in the aquaculture industry.

## 1. Introduction

Microalgae, as the name implies, are unicellular microscopic algae, which, depending on the species, can range from a couple of micrometres (µm) to a few hundred µm in size. They are typically found individually as well as in chains or groups in freshwater and marine systems [1]. Due to their nutritional values, microalgae have long been proposed as live feed in the aquaculture industry [2]. Among them, indigenous microalgae are widely preferred and utilized due to their high growth rate and robustness in survivability even when exposed to unpredictable and erratic tropical weathers [3]. Their lack of cell wall is also advantageous as it allows larvae, the juvenile form of animals, to easily digest and assimilate microalgae into their system [4,5,6]. Besides aquatic organisms, human beings can also benefit from supplementing the diet with microalgae as several studies have testified their health benefits in boosting the production of red blood cells along with their excellent performance as antioxidant, anti-inflammatory, anti-mutagenic, and antimicrobial agents [7].

Microalgae represent the first component of the aquatic food chain as they are a type of photosynthetic microorganism. This suggests their enormous importance and potential in generating abundant aquaculture yield by manipulating the food web causal relationship [8,9,10]. For instance, they are frequently used in the hatcheries of several aquatic animals, such as for some fish species at their primary stage of development, certain crustacean species at larval stage, various stages of bivalves (broodstock, larvae, juveniles), and post-set abalone, and as the main feeding material for zooplankton [11,12,13]. Being a natural food in the ecosystem, besides having significantly high nutritional values, makes microalgae a suitable choice of immunostimulants to be incorporated into the feed [14] as showcased by the positive effects of species such as *Chlorella vulgaris* and *Spirulina platensis* on the immunological parameters (lysozyme activity, phagocytic activity, complement activity, respiratory burst activity, and increased plasma protein) of certain fishes in multiple studies [15,16,17].

*Isochrysis galbana*, a golden-brown and flagellated marine microalga [18], is rich in polyunsaturated fatty acids (PUFA) such as docosahexaenoic acid (DHA) and eicosapentaenoic acid (EPA) [19] and is a valuable source of photosynthetic pigments like chlorophyll a and fucoxanthin [20], two abundantly available bioactive resources among microalgae [21,22,23,24]. Previous phytochemical studies on *I. galbana* selectively focused on carotenoids [25], fatty acids [26,27] and triacylglycerols [28]. Aguilera-Sáez et al. (2019) [29] recently performed an NMR-based metabolomics study on *I. galbana* in which amino acids, lipids, and sterols were profiled. These studies emphasised that the selection of solvents used for extraction is vital for the recovery of metabolites, a crucial stage in the workflow of a metabolomics analysis to capture the broadest number of metabolites possible [30].

Nuclear Magnetic Resonance (NMR) Spectroscopy and Liquid Chromatography-Mass Spectrometry (LCMS) are the two most common and robust analytical platforms used in metabolite profiling studies [31,32,33]. The advantages of NMR spectroscopy as an analytical tool include simple sample preparation, fast data acquisition, and high reproducibility [34,35]. On the other hand, LCMS offers other complementary features from its high sensitivity in metabolite detection (up to picomolar level), few problems with peak overlaps, and high reliability in the identification of different metabolites from a sample. Bearing in mind that there is no single analytical platform that can perform a complete identification and quantification of all molecules within a sample [36], the combination of these two powerful spectroscopic tools is certainly advantageous for the coverage, sensitivity, and reliability of the generated results. In addition, molecular networking (MN) has been an important bioinformatics platform to simulate and annotate untargeted mass spectrometry (MS) data [37,38] since its launch in 2012 [39]. This approach offers new ways of exploring the metabolome of biological samples by providing essential analog knowledge among the metabolites identified. Numerous MN applications in microalgae chemistry have been documented in recent years [40,41,42].

Hence, in this study, NMR fingerprint and LCMS profile of *I. galbana* extracted in five different solvents with different polarities were performed to determine the best solvent that can extract a wide range of metabolites in high concentrations. The results produced can pave the way towards developing a standardized biomass or extract from this microalgae species. To the best of our knowledge, there have been no studies done on the metabolite profiling of *I. galbana* using NMR in combination with LCMS as described herein.

## 2. Results and Discussion

### 2.1. Identification of Metabolites in NMR Spectra of Different Solvent Extracts

**Figure 1:**
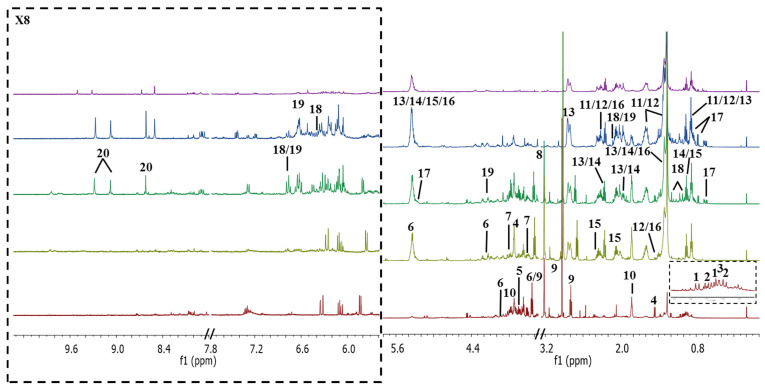
Representatives 1D 500 MHz ^1^H NMR spectra of hexane (Hex), ethyl acetate (EtOAc), absolute ethanol (EtOH), 50% ethanol (AqE), and aqueous (Aq) extracts of *I. galbana* dissolved in CD_3_OD:CDCl_3_ in the ratio of 8:2, with 0.05% trimethylsilylpropanoic acid (TSP) as an internal reference standard. The assignments of the peaks are listed in Table 2.

Figure 1 displays the representatives 1D 500 MHz ^1^H NMR spectra of *I. galbana* in Hex, EtOAc, EtOH, AqE and Aq extracts. The individual spectrum of each solvent extract was provided in the Supplementary File (Figure S6). Overall, the Aq spectrum showed fewer metabolite signals with low intensity as compared with the other solvents. This might be due to its low dissolution in the NMR solvent used (CD_3_OD:CDCl_3_ in the ratio of 8:2), unlike the other extracts. However, the yield of Aq extract was the highest (Table 1), while Hex extract gave the lowest yield which, suggests that *I. galbana* contains more polar metabolites than the less and non-polar ones. In the aromatic region, the signals were intense in EtOAc extract followed by EtOH and Hex extracts. Further interpretation of the assigned signals was completed using ^1^H NMR, while 2D-J-RES and HSQC experiments were utilized in order to increase the metabolites specificity and to minimize the congestion of the signals. The peaks were assigned by referring to previous studies [29,43,44,45] and by comparing them with freely available online databases such as Human Metabolome Database (HMDB) and PubChem.

A total of 21 metabolites from different chemical classes were putatively identified along with their chemical shift assignments, corresponding to multiplicities and coupling constants values as summarized in Table 2. The upfield region of the spectrum (0.8 to 3.0 ppm), which belongs to the aliphatic compounds, was mostly contributed by amino acids and fatty acids [47]. The five amino acids, which were identified as valine, isoleucine, leucine, alanine, and threonine, were clearly detected in the polar solvent, particularly in Aq extract as presented in Figure 1. These metabolites were previously reported in the same microalgae species [29] and also in another species known as *Pleurochrysis carterae*, which belongs to the same Haptophyta phylum as *I. galbana* [44].

**Figure 2:**
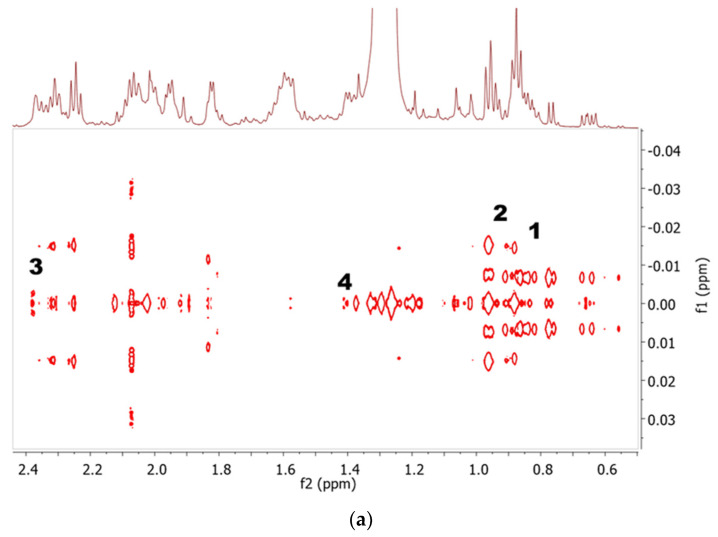

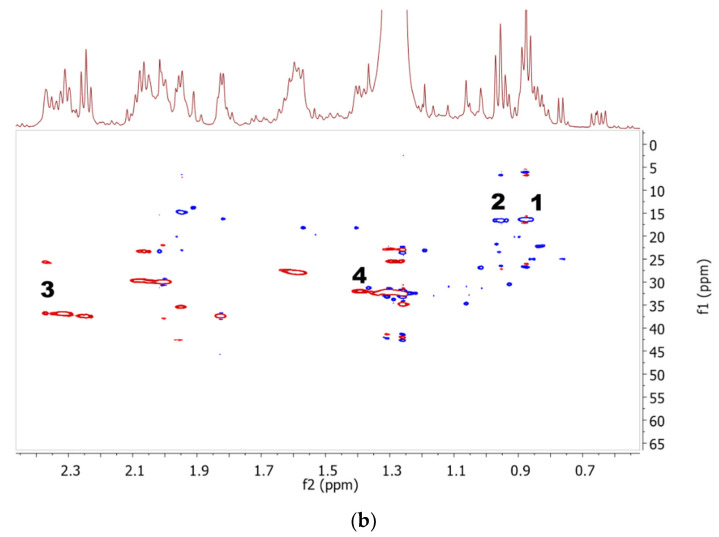
Representative 2D-J-resolved (**a**) and HSQC spectra (**b**) of EtOAc extract. The signals were assigned as follows: 1, non-PUFA; 2, PUFA; 3, DHA; and 4, DPA.

The upfield region of all spectra, with the exception of the Aq extract, exhibited signals for both saturated and unsaturated fatty acids from the appearance of characteristic terminal methyl protons at 0.88 ppm. This signal was correlated to the last carbon attached to the methyl group at 16.4 ppm in the HSQC spectra. Meanwhile, the presence of PUFA with omega-3 FAs was confirmed with the triplet signal observed at 16.6 ppm from the terminal methyl protons of its last carbon and the HSQC cross-peaks for the carbon at 16.6 ppm [29], as shown in Figure 2a. The presence of saturated palmitic acid (C16:0) and arachidic acid (C20:0) was identified from the multiplet at 1.33 and 1.40 ppm, respectively. The unsaturated fatty acids were verified as oleic acid (18:1), α-linoleic acid (C18:3), docosahexaenoic fatty acid chain (DHA) (C22:6), and docosapentaenoic fatty acid chain (DPA) (22:5). The characteristic signal for DHA was assigned at 2.38 ppm, and it appeared as a small triplet which HSQC cross-peaks to a carbon (C-2) at 36.8 ppm in the HSQC spectra. This observation is in agreement with a study by Aguilera-Sáez et al. (2019) [29]. Another PUFA was assigned as docosapentaenoic fatty acid chain (DPA) from the multiplet peak at 1.38 ppm, which cross-peaks to C-20 at 31.2 ppm in HSQC. The J-resolved and HSQC spectra for both PUFAs (DHA and DPA) are displayed in Figure 2b. In previous studies, *I. galbana* was reported to contain variable levels of eicosapentaenoic fatty acid (EPA) [48,49]. However, EPA was not detected in the present study, which might be because of the high concentration of PUFAs. Culture conditions can highly affect the concentration of PUFAs [4], which may be the possible reason for the undetected EPA in this study. The level of EPA also depends on the elongation of EPA into DPA before subsequent desaturation into DHA by Δ6 desaturase occurs in the pathway of omega-3 long-chain-PUFA biosynthesis [50]. Another discovered metabolite, which was cholesterol, exhibited sterol signals in the region of 0.62–0.68 ppm, which is again similar to what was reported by Aguilera-Sáez et al. (2019) [29]. Its characteristic doublet of doublet signal was observed at 5.28 ppm after being compared with an online database.

**Figure 3:**
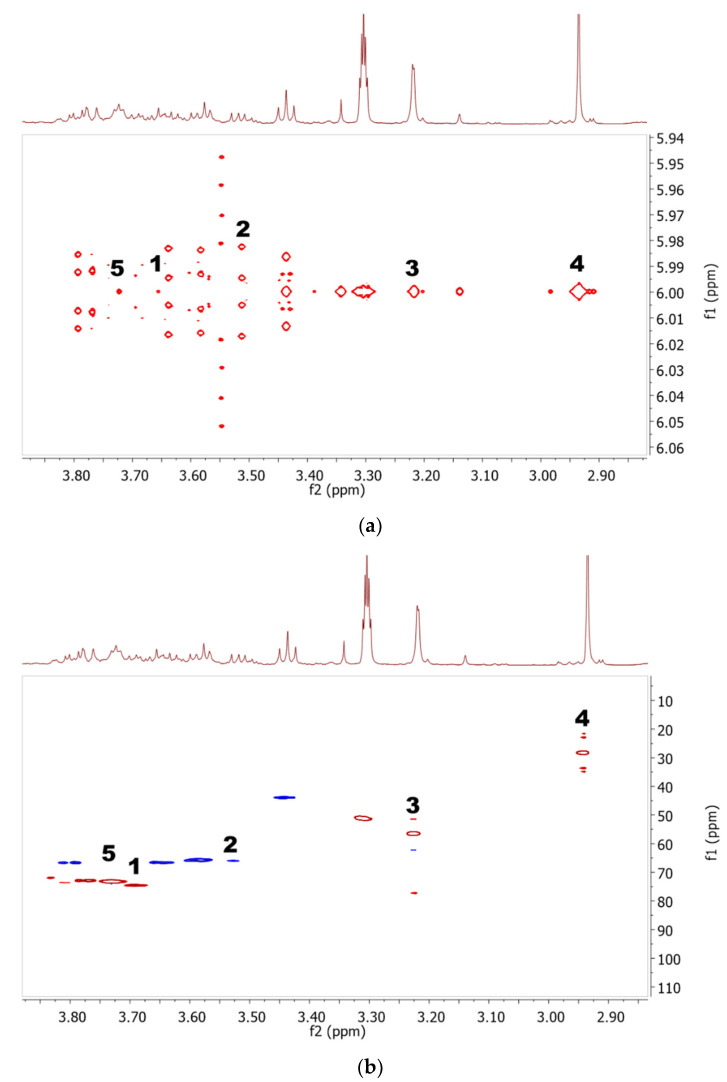
2D-J-resolved (**a**) and HSQC spectra (**b**) of Aq extract in the region 2.9 to 3.8 ppm. The signals were assigned as follows: 1, sucrose; 2, glucose; 3, choline; 4, DMSP; and 5, d-1,4/2,5-cyclohexanetetrol.

The middle region of the spectra (3.5 and 5.5 ppm) was congested with signals of sugars, including carbohydrates, and other groups of metabolites that were discovered in Aq extract. Confirmation of the assigned metabolites was aided by information from 2D J-resolved and ^1^H-^13^C HSQC correlation, as displayed in Figure 3. These metabolites were mainly observed in the polar extracts (Aq, AqE, and EtOH). The presence of sucrose was justified by a characteristic singlet at 3.68 ppm, which cross-peaked to C-3 of cyclohexane ring at 74.5 ppm. Meanwhile, glucose was assigned based on the anomeric proton doublet of doublet peak at 3.52 ppm with its HSQC correlation to C-7 (66.5 ppm) attached to a hydroxyl group [45]. Choline, another primary metabolite found, was observed as a singlet at 3.22 ppm with its HSQC correlation to 57.0 ppm for its three methyl substituents attached to an amino skeletal [29]. The rest of the metabolites detected in this region were dimethylsulphoniopropionate (DMSP) and d-1,4/2,5-cyclohexanetetrol, which were putatively identified based on the characteristic singlet at 2.92 ppm and multiplet at 3.72 ppm with the correlation of HSQC to carbons at 28.3 ppm and 73.5 ppm (C-1,2,4,5) respectively, comparable to those reported by Aguilera-Sáez et al. (2019) [29].

The downfield region from 6.0 to 8.5 ppm displayed signals of aromatic resonances from the carotenoids and chlorophyllic constituents, specifically the olefinic protons (5.8–6.8 ppm), which had a few signals in the aliphatic region (0.8–2.5 ppm) [51]. These observations allowed the identification of several carotenoids, with much complications due to their small differences in structure; for example, the identification of fucoxanthin and violaxanthin was difficult and confusing due to the similarity in their olefinic chains. However, the presence of fucoxanthin was justified by the singlet of the terminal methyl protons at 1.37 ppm and 2.12 ppm for the cycloalkane moiety, which were not present in violaxanthin. Meanwhile, the presence of another carotenoid, astaxanthin, was confirmed via HSQC crossed-peak between proton signal at 4.15 ppm to the corresponding carbon (65.2 ppm) attached to a hydroxyl group. These carotenoids were mostly detected in the organic solvent extracts of EtOAc and EtOH, as displayed in the stacked spectra of ^1^H NMR (Figure 1). Another pigment identified as chlorophyll could be clearly spotted in the spectra from the isolated proton signals between 8.5 to 11.2 ppm [52]. In this study, all singlets that appeared between 8.60–9.21 ppm in the non-polar extracts were assigned as pheophytin a. Correlation with the nearest carbon between 96.2–107.2 ppm in HSQC spectra confirmed their identification, as previously reported [47].

Including *I. galbana*, there are four microalgae strains of the haptophyte in the order of Isochrysidales, which contain polyunsaturated long-chain alkenones (PULCAs) [53]. These unsaturated ketones are made up of a carbon chain of C37–C39 with 2 to 4 *trans*-type double bonds, which usually present at the intervals of seven carbon atoms [54]. Recently, Richter et al. (2019) [55] found C37–C39 alkenones in *I. galbana*. In the current study, the alkenones were characterized by the appearance of singlet methyl protons at 2.12 ppm. Meanwhile, the presence of *trans*-type double bond was confirmed by the signals of the allylic proton, which attached to carbon with a double bond at 1.95 ppm and crossed-peak to carbon at 35.4 ppm in the HSQC spectra, as reported by Iglesias et al. (2019) [46].

Overall, the 21 metabolites, including amino acids, carbohydrates, fatty acids, carotenoids, and chlorophyll, were successfully determined using NMR spectroscopy. These results verified the convenience of NMR in metabolomics profiling studies as almost all metabolites from different classes of compounds were detected in just one type of analysis [56]. Unfortunately, there is a limitation in using a single deuterated solvent: the combination of CD_3_OD and CDCl_3_ (8:2) was able to completely dissolve most of the extracts but not for Aq and Hex extracts due to the difference in polarity. Hence, this hindered the detection of the maximum number of compounds, as reported in other studies [29,46]. The selection of NMR solvent system was based on a preliminary optimization step using several different solvents combinations. The microalgae extracts were found to dissolve better in CD_3_OD and CDCl_3_ (8:2) solvent system as compared to the others. The metabolites detected in the present study cover a wide range of compound classes. Furthermore, it is mandatory to use the same solvent system in both NMR acquisition and data bucketing/binning for multivariate data analysis. All spectra were aligned and binned together before the solvent regions in each spectrum were uniformly excluded. Using a different solvent system for each solvent extract may seem wise so as to achieve high solubility; however, this will bring about an inconsistency during data processing for multivariate data analysis.

### 2.2. Discriminative Analysis of Solvent Extracts Based on ^1^H NMR Data

Partial least square-discriminant analysis (PLS-DA) was applied to classify the predefined groups of *I. galbana* solvent extracts and generate information on the discriminating metabolites among the groups [57]. This technique is a supervised method, requiring class label information in building the appropriate model for data interpretation. A model is interpreted as good if its cumulative R2 and Q2 values are close to 1, with R2 giving an overview of the model fitness, while Q2 describes the predictive quality of the model [58]. The validity of the models is evaluated using a permutation test (100 permutations), wherein, for a valid model, the R2 and Q2 intercepts should not exceed 0.3–0.4 and 0.05, respectively [58].

**Figure 4:**
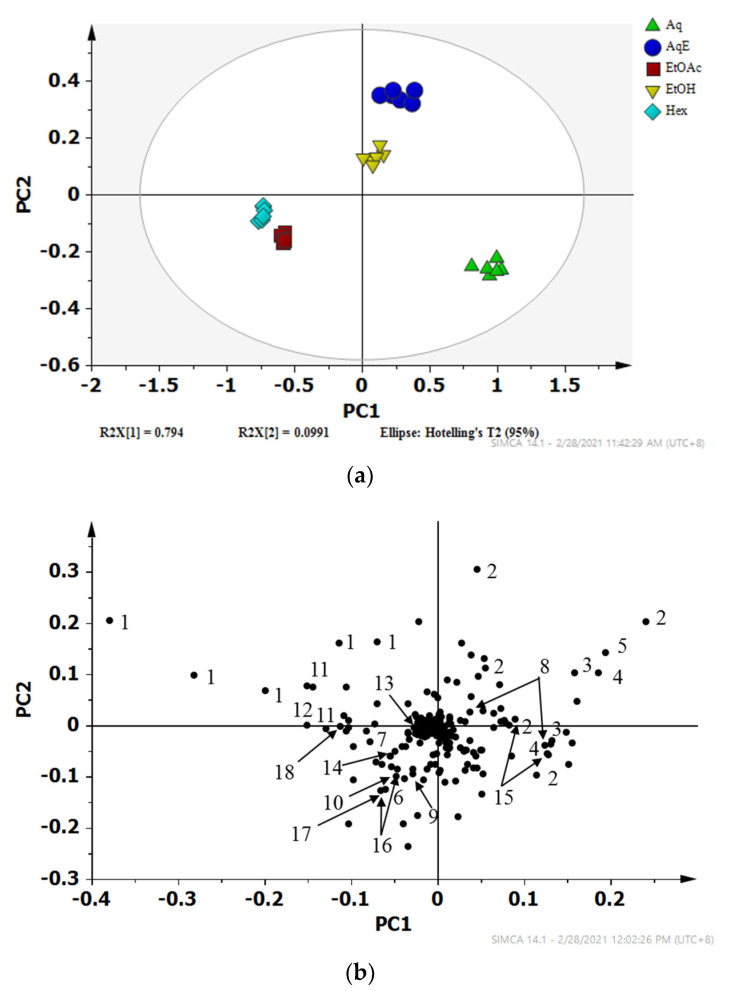
PLS-DA (**a**) scores and (**b**) loading scatter plots of the ^1^H NMR data representing *I. galbana* extracted in 5 different solvents; Aqueous (Aq), 50% ethanol (AqE), ethanol (EtOH), ethyl acetate (EtOAc) and hexane (Hex). Corresponding metabolites with VIP value more than 1:1, fatty acid derivatives; 2, DMSP; 3, d-1,4/2,5-cyclohexanetetrol; 4, amino acid derivatives; 5, choline; 6, valine; 7, threonine/DPA/fucoxanthin; 8, sucrose; 9, archidic acid; 10, leucine/*α*-linoleic acid; 11, palmitic acid; 12, *α*-linoleic acid; 13, astaxanthin; 14, cholesterol; 15, glucose; 16, carotenoid derivatives; 17, sucrose/DHA/DPA; 18, oleic acid.

The PLS-DA score plot (Figure 4a) shows that the Aq, AqE, and EtOH extracts were discriminated from the EtOAc and Hex extracts by principal component (PC) 1. The R2 and Q2 values were 0.98 and 0.97 respectively, indicating a model of good fit, while the R2 and Q2 intercepts were between 0.21 and −0.49, respectively, further proving the robustness of this model (see Supporting Information Figure S1). Meanwhile, the metabolites that were responsible for the separation could be identified from the loading plot (Figure 4b) with their respective chemical shift binned to the width of 0.04 ppm. The metabolites such as DMSP, d-1,4/2,5-cyclohexanetetrol, choline, sucrose and glucose that were located on the positive side of PC1 are more prominent in Aq, AqE, and EtOH extracts, while those on the negative side of PC1 such as fatty acids and carotenoids were higher in EtOAc and Hex extracts. The importance and significance of the variables were determined by analysing the Variable Importance in Projection (VIP) plot with jack-knifing uncertainty bars. The variables with VIP scores greater than 1 with an error bar not crossing the baseline in the loading column plot were retained as significant and were classified as chemical markers that gave an influential contribution to the discrimination in the PLS model [59]. Based on their VIP values being greater than 1.0, thirty-two binned regions, as illustrated in the VIP plot (see Supporting Information Figure S2), significantly contributed to the class separation. Some metabolites were assigned based on several corresponding binned regions, for example, five NMR peaks (2.7, 2.9, 2.94, 3.42 and 3.46) for DMSP alone.

### 2.3. Relative Quantification

**Figure 5:**
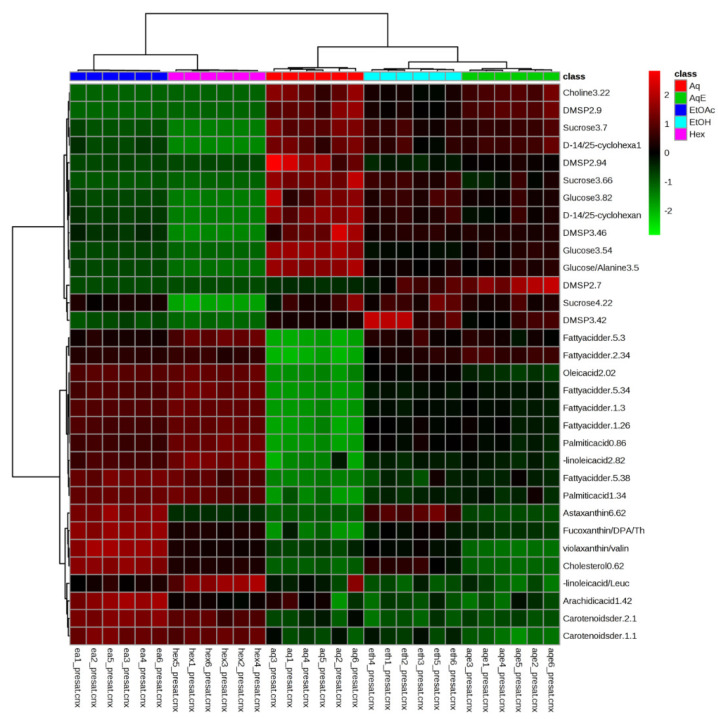
Heat map of identified metabolites of *I. galbana* extracted in 5 different solvents—Aqueous (Aq), 50% ethanol (AqE), ethanol (EtOH), ethyl acetate (EtOAc), and hexane (Hex). The concentration of each metabolite is colored based on a normalized scale from minimum−3 (dark green) to a maximum of 3 (dark red).

In order to view the distribution of the identified metabolites among different solvent extracts, the relative concentration of the metabolites was analysed and visualized using HCA. The binned regions with VIP values greater than 1 were selected from the original data set, normalized with Pareto scaling and subjected to HCA using Euclidean distance measures and Ward’s clustering algorithm. The results of the analysis were visualized as a color-coded heat map (Figure 5), which helps to reflect the distribution and relative intensities of the metabolites.

The heat map indicated that EtOAc and Hex extracts contained higher concentrations of fatty acids and carotenoids as compared to the other extracts. The Aq extract had the highest levels of DMSP, D-1,2,4,5-cyclohexanetetrol, choline, sucrose, and glucose, followed by the AqE and EtOH extracts. The heat map was in agreement with the discrimination of metabolites seen in the PLS-DA loadings plot (Figure 4b). Moreover, most of the putatively identified carotenoids and unsaturated fatty acids were discriminated on the negative side of PC1, verifying the high intensity (concentration) of these metabolites in EtOAc and Hex extracts.

The heat map also shows that the technical replicates of the same origin were grouped closely together, indicating a good reproducibility in both the sample extraction and ^1^H NMR measurement. The HCA dendrogram shows the samples are divided into two distinct clusters comprising EtOAc and Hex extracts in one and Aq, AqE, and EtOH extracts in another. The EtOH and AqE extracts were further grouped together and separated from the Aq extract. The only difference between EtOAc and Hex extracts was the intensity of carotenoids (fucoxanthin and astaxanthin), wherein these compounds were higher in EtOAc than Hex based on the colour intensity in the map. Hence, the HCA and PLS-DA results strongly suggest that ethyl acetate (EtOAc) is the best solvent to be used in order to extract most of the valuable fatty acids and carotenoids in high concentration from *I. galbana*.

To further validate the results, important metabolites were relatively quantified based on the peak area values of their characteristic chemical shifts before being analysed using two-way ANOVA test (with a threshold of *p* < 0.05). Out of the thirty-two metabolites identified from the VIP plot, only 10 compounds were deemed to be important in causing the group separation as their concentrations varied in a statistically significant manner. As for quantification, only peaks that did not overlap with other peaks and referred to a single metabolite were selected, such that at 3.66 ppm (s) for sucrose, 3.54 ppm (dd) for glucose, 3.22 ppm (s) for choline, 2.90 ppm (s) for DMSP, 1.82 (m) for d-1,4/2,5-cyclohexanetetrol, 1.42 ppm (m) for arachidic acid, 1.34 ppm (m) for palmitic acid, 2.82 ppm (m) for α-linolenic acid, 0.66 ppm (m) for cholesterol, and 6.62 (d) for astaxanthin. The derivatives of the carotenoids and fatty acids were excluded from the quantification process since these metabolites do not clearly display any characteristic peaks.

**Figure 6:**
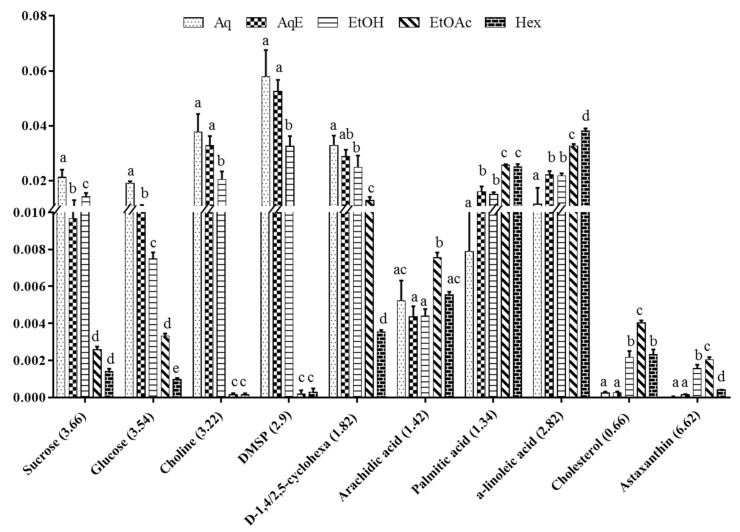
Relative quantification based on the mean peak area of ^1^H-NMR signals of identified metabolites in *I. galbana* extracted in 5 different solvents: Aqueous (Aq), 50% ethanol (AqE), ethanol (EtOH), ethyl acetate (EtOAc), and hexane (Hex). Data are expressed as a mean of six replicates each of the solvent systems ± standard deviation (SD). Means with different letters indicate significant differences (*p* < 0.05; *n* = 6).

Relative quantification of the selected metabolites was presented in a bar chart along with their statistical difference (Figure 6). There was a similar trend among the different extracts with regard to the quantification of sucrose, glucose, choline, DMSP, and d-1,4/2,5-cyclohexanetetrol. The more polar extracts, particularly the Aq, followed by AqE and EtOH, demonstrated a high significance difference (*p* < 0.05) for these five metabolites. Sucrose, glucose, and d-1,4/2,5-cyclohexanetetrol were also found in EtOAc extract at 85.7%, 84.2%, and 60.6%, which were lower than that in the Aq extract. A study on Ajwa dates reported the same observations, whereby simple sugars such as sucrose and glucose, are better extracted by polar solvents like methanol at concentrationss of 50% and 100% [60]. Choline and DMSP, on the other hand, were not obvious in EtOAc and Hex extracts due to the very small signal-to-noise ratio.

Nevertheless, another 5 compounds from the class of fatty acids and carotenoids, namely arachidic acid, palmitic acid, α-linolenic acid, cholesterol, and astaxanthin, were observed the highest (*p* < 0.05) in EtOAc extract. Comparing between both the EtOAc and Hex extracts shows that arachidic acid and cholesterol were higher in the former with concentrations of 36.4% and 69.2%, respectively, while α-linolenic acid was higher in the latter, with the concentration of 18.9%. The effect of different solvents on the efficacy of extracting fucoxanthin was previously investigated, and ethyl acetate was shown to be more efficient than chloroform and ethanol [61]. In another study, the chloroform extract of *Chaetoceros calcitrans* was shown to give a higher amount of palmitic acid and cholesterol, which suggests that moderately polar solvents like ethyl acetate and chloroform are the best for fatty acids extraction [45]. Most of the fatty acids were readily detected in the Aq and AqE extracts. Thus, results from PLS-DA, HCA, and relative quantification established that EtOAc is the best solvent to extract high amounts of fatty acids and carotenoids, while amino acids and carbohydrates are more efficiently extracted using water. Hence, EtOAc and water were selected as the most suitable solvents for the recovery of high concentrations of metabolites from *I. galbana*, as it is crucial to obtain a wide range of compounds in the highest intensity possible [30]. This result is in agreement with the previous study on fucoxanthin, which was extracted efficiently in EtOAc solvent when compared with the other solvents [62].

In the extraction process, solvents used must have the same polarity as the solute of interest in order to effectively dissolve the targeted metabolites [63]. Different classes of solutes need certain types of extraction solvents since their chemical properties can be in different polarity and can also be thermally unstable. The extraction process is a crucial initial stage to ensure the bioactive compounds in a medicinal plant are preserved prior to further analysis [64]. The principle of solvent selection relies most significantly on the specific characteristics of the targeted compound(s) to be isolated [64]. In the present study, most of the bioactive compounds reported in *I. galbana* such as polyunsaturated fatty acids (PUFA), docosahexaenoic acid (DHA), and photosynthetic pigments like chlorophyll a and fucoxanthin [20] were observed to be highly present in EtOAc extract.

### 2.4. UHPLC–MS/MS Analysis

**Figure 7:**
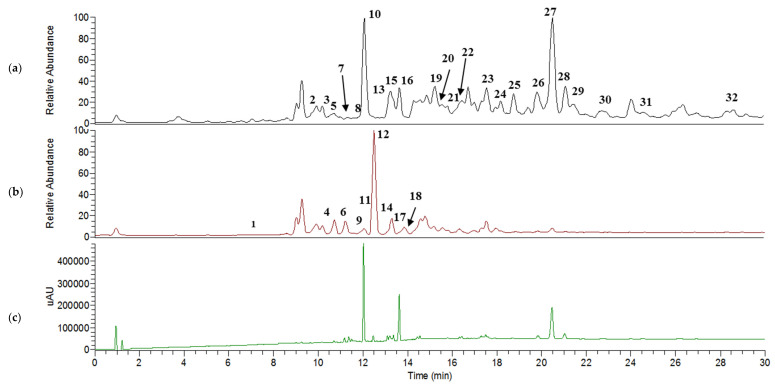
Total ion chromatogram (TIC) of *I. galbana* EtOAc extract analysed in positive mode (**a**), negative mode (**b**), and PDA (photodiode array) (**c**).

LCMS had been previously used to identify fatty acids, triacylglycerols, and carotenoids in *I. galbana* [25,28]. However, the system used was limited on the scan range and used atmospheric pressure chemical ionization (APCI) as the ionization method instead of electrospray ionization (ESI). In the present study, a UHPLC system coupled with Q-Exactive Focus Orbitrap LCMS/MS, which is a more advanced system known for its higher sensitivity and excellent mass accuracy, was utilized to achieve a more comprehensive metabolite profile of this microalgae. The total ion chromatogram (TIC) (Figure 7a,b) shows the peaks detected in the positive and negative ion modes for EtOAc extract. This extract was selected for further analysis since its NMR data showed most of the metabolite groups in high concentrations. The mass analysis was conducted in switching mode [65,66]. Using both positive and negative ionization modes to identify compounds enables a broader coverage of the metabolome than the use of a single polarity [67,68], as some analytes can only be detected in either one of these modes. The spectral interpretation software predicts and automatically generates detailed fragmentation according to the general principles of ionization, fragmentation, and rearrangement using chemical structure provided by databases such as HMDB and PubChem.

#### Metabolite Identification in Positive and Negative Ion Mode

The identity, retention time, UV characteristic, and fragment ion(s) for each metabolite are presented in Table 3, and the MS/MS spectra for every molecular ion detected are available in the Supporting Information (Figure S3). Thirty-two metabolites comprising eight carotenoids, five chlorophylls, four glycerophospholipids, three sphingolipids, one glycerolipid, fatty acid, and sterol were annotated in the positive mode. For the negative mode, nine main peaks were characterized as fatty acids. All metabolites detected showed high mass accuracy with their scored mass error being less than 10 ppm. However, both arachidic acid and cholesterol displayed high mass error values (>400 ppm), indicating poor accuracy as compared with their theoretical values. Nonetheless, these two metabolites’ fragments, which were ambiguously matched with the HMDB database were positively detected in NMR. Most of the carotenoids were discovered in the photodiode-array (PDA) spectrum (Figure 7c). The identification of fucoxanthin as a major carotenoid with a small mass error value was found in the positive mode at *m/z* 659.4283 [M + H]^+^ and at t_R_ 12.07 min of the chromatogram by comparing with PubChem database, whereas the fragments of *m/z* 581, 411, 355, and 199 were matched with Massbank database.

**Figure 8:**
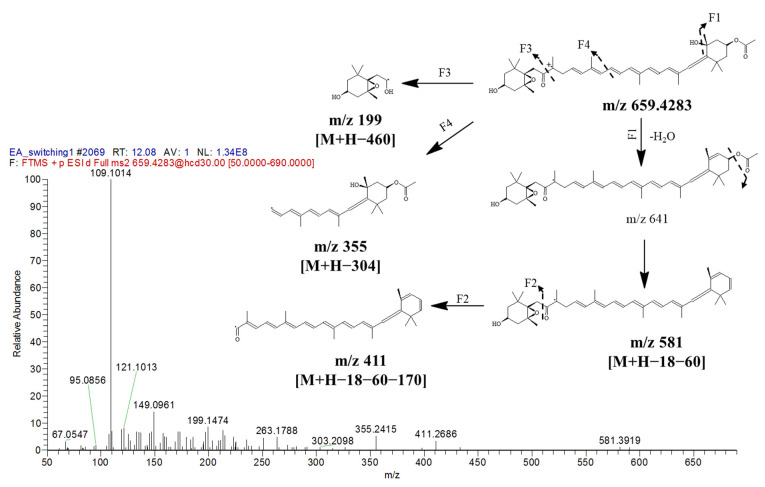
MS/MS spectrum of fucoxanthin at [M + H]^+^ *m*/*z* 659.4283 with proposed fragmentation patterns.

The series of fragmentation pattern was proposed in Figure 8, starting with fragment 1 (via F1), which displayed the loss of H_2_O molecule from the parent ion, and this was followed by the inductive cleavage of the carboxylic group to yield *m/z* 581. The second fragmentation (F2) was suggested due to the cleavage at the carbon-oxygen bond in *m/z* 581 to yield *m/z* 411. Besides this, there were two other fragmentations (F3 and F4) that occured from the cleavage of the carbon-carbon bond of the long carbon chain and generated *m/z* 199 [M + H − 460]^+^ and *m/z* 355 [M + H − 304]^+^, respectively. These results are in agreement with the NMR data, which identified fucoxanthin as the main carotenoid in EtOAc extract.

**Figure 9:**
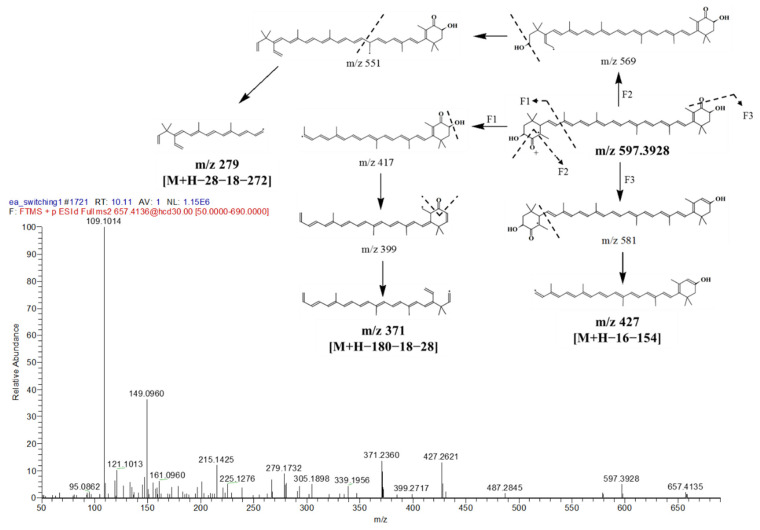
MS/MS spectrum of astaxanthin at *m*/*z* 657.4136, [M + IsoProp + H]^+^ and proposed fragmentation patterns.

Astaxanthin (Figure 9) was detected at t_R_ 10.11 min with isopropanol adduct [M + IsoProp + H]^+^ exhibiting its ion at *m/z* 657.4136 [M + 61]^+^. Some characteristic MS fragments observed were matched with the Massbank database of astaxanthin. The first fragment (F1: Figure 9) arose from the inductive cleavage at carbon bond to produce *m/z* 417. This was followed by a loss of an H_2_O molecule, due to the hydrogen being attacked by a hydroxyl, and the cleavage of CO moiety, which resulted in the ion at *m/z* 371 [M + H − 180 − 18 − 28]^+^. A second fragment via F2 was derived after the loss of CO moiety and H_2_O and before the inductive cleavage at the long carbon chain yielding ion *m/z* 279 [M + H − 28 − 18 − 272]^+^. The next fragmentation (F3) was due to the loss of oxygen from the hydronium ion formation plus the cyclohexyl group [M + H − 16 − 154]^+^ ion cleavage, which resulted in *m/z* 427. Another major carotenoid, phoenicoxanthin, was detected at t_R_ 13.63 min with its [M + H]^+^ ion at *m/z* 581.3964. This carotenoid was reported in *Aurantiochytrium* sp., a colorless fungus-like algae, which contains a similar composition of carotenoids and PUFAs as *I. galbana* [69].

Another major fatty acid was putatively identified as DHA at 12.49 min with a small mass error (−0.31 ppm) from the theoretical mass in the PubChem database. The presence of DHA, DPA, *α*-linolenic acid, palmitic acid, arachidic acid, and oleic acid was justified by the NMR results. Four additional acids, namely EPA, hexacosanedioic acid, 3,6,9,12,15-octadecapentaenoic acid, and stearidonic acids, which were not identified in the NMR results, were detected in the mass data. These fatty acids had been previously reported in the marine haptophytes, dinophytes, and prasinophytes [70] and are one of the main PUFAs found in *Isochrysis zhangjiangensis* [71].

**Figure 10:**
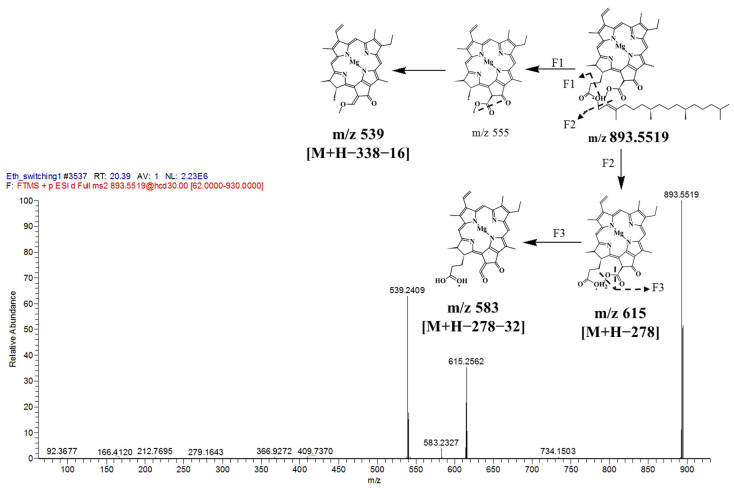
MS/MS spectrum of chlorophyll a with *m*/*z* 893.5519, [M + H]^+^ and proposed fragmentation patterns.

Chlorophyll a was detected in the positive mode with an ion at *m/z* 893.5519 (t_R_ 20.39 min). The fragmentation pattern of F1 (Figure 10) suggested an inductive cleavage at the long carbon chain, which yielded *m/z* 555, followed by the loss of oxygen at carbonyl group, which produced *m/z* 539 [M + H − 338 − 16]^+^. The second fragment (F2) was derived due to cleavage of a long carbon chain to yield *m/z* 615. Subsequent fragmentation occurred from *m/z* 615 with the loss of methoxy group (32 amu), giving an ion at *m/z* 583 [M + H − 278 − 32]^+^. This observation is in agreement with a previous large-scale microalgae production study that identified chlorophyll a as the main chlorophyll of *I. galbana* [72]. In another study, Crupi et al. (2013) [73] detected pheophytin a, one of the main pigments in *I. galbana*, by using HPLC-DAD-MS.

### 2.5. MS/MS-Based Molecular Networking

Molecular networking facilitates a fast comparison of mass spectrometry profiles from complicated crude extracts for successful metabolites dereplication and exploration of novel compounds, which requires a high resolution of mass spectrometry data (MS/MS) [39]. Dereplication is defined as the fast detection of defined metabolites through the comparison of experimental mass spectra with libraries. All metabolites are represented as parent ions, which are linked by the chemical fragmentation of the compound. Related compounds comprised similar parent ion fragmentation patterns, which are represented as a cosine score from 1 (extremely similar fragmentation spectra) to 0 (totally different parent ions) [39,74]. Therefore, the parent ions (nodes) are bound by edges with cosine score value, resulting in the classification of analogous or structurally related compounds in molecular clusters [39,75].

**Figure 11:**
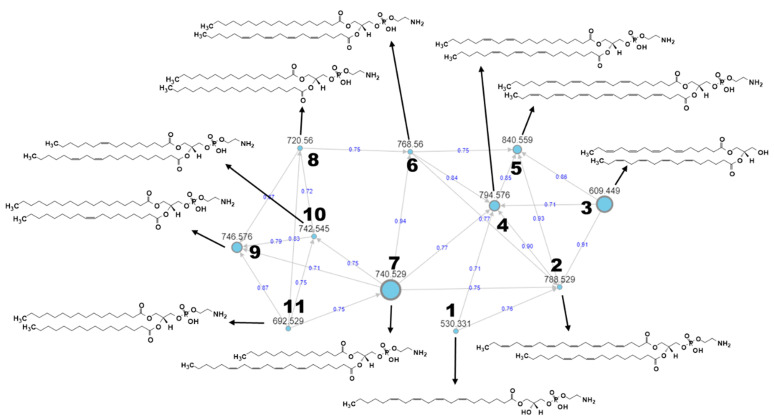
MS/MS-based molecular networking from a cluster of glycerophospholipids and identifying 11 new structures as listed in Table 4. Nodes are labeled with parent *m/z* values, with different size corresponding to precursor intensity. Edges are labeled with cosine scores from 0 to 1.

In the present study, a classical global molecular network was generated based on UPLC–MS/MS data from EtOAc extract with blank using the GNPS platform in order to focus on the lipid content of *I. galbana* more comprehensively as microalgae become one of the promising sources of lipid production [76]. The putative identified metabolites were achieved by manual dereplication matched with several external databases, namely HMDB, PubChem, LIPID MAPS, and Chemspider through Metabolomics Workbench platform (www.metabolomicsworkbench.org, accessed on 28 October 2020) with the lowest mass error since automated dereplication on the GNPS platform was limited and did not match any known compound. Overall, there were thirteen clusters, generated with more than two nodes per cluster as shown in the Supplementary File (Figure S5). However, most of the clusters were not fully annotated due to the limited databases search, except for one of the largest clusters, which was identified as families of glycerophospholipids and consisted of eleven putative metabolites as shown in Figure 11. Other glycerolipids such as monogalactosyldiacylglycerols (MGDG) and digalactosyldiacylglycerols (DGDS) might be identified from other clusters if other databases are explored. This will be a time-consuming task that should be thoroughly taken up in future studies. 

Glycerophospholipids are made up of phosphatidic acids, compounds constructed by a glycerol molecule with fatty acid esterification in two of its hydroxyl groups and phosphoric acid esterification in the third hydroxyl. Commonly, one of the phosphate moieties’ free OH groups is esterified with another element, forming various glycerophospholipids [77]. In the current cluster, ten of them are classified as phosphatidylethanolamine (PE), since a glycerol replacement site is dominated by the phosphorylethanolamine moiety as referred to in Table 4. Unlike the others, the first metabolite, known as lysophospholipid, refers to a phospholipide that is lacking one of the two *O*-acyl chains. Like diacylglycerols, PE can have several combinations of fatty acids with differing lengths and saturation at positions C-1 and C-2, with the most popular are fatty acids containing 16, 18 and 20 carbons. The third structure is annotated as diglyceride (DG), consisting of two fatty acid chains, which are stearidonic acid at the C-1 and C-2 positions covalently bonded to a glycerol molecule through ester linkages without phosphorylethanolamine moiety. This finding has proven the existence of several phospholipids assigned as PE and phosphatidylcholine (PC) in the lipophilic extracts of *I. galbana* detected by two-dimensional ^1^H−^31^P HSQC TOCSY (heteronuclear single quantum coherence-total correlation spectroscopy) [29]. Moreover, the presence of PE can be observed significantly in other species of microalgae such as *Chaetoceros gracilis, Nannochloropsis gaditana* and *Picochlorum atomus,* especially after three days of cultivation under phosphate repletion study [78].

**Figure 12:**
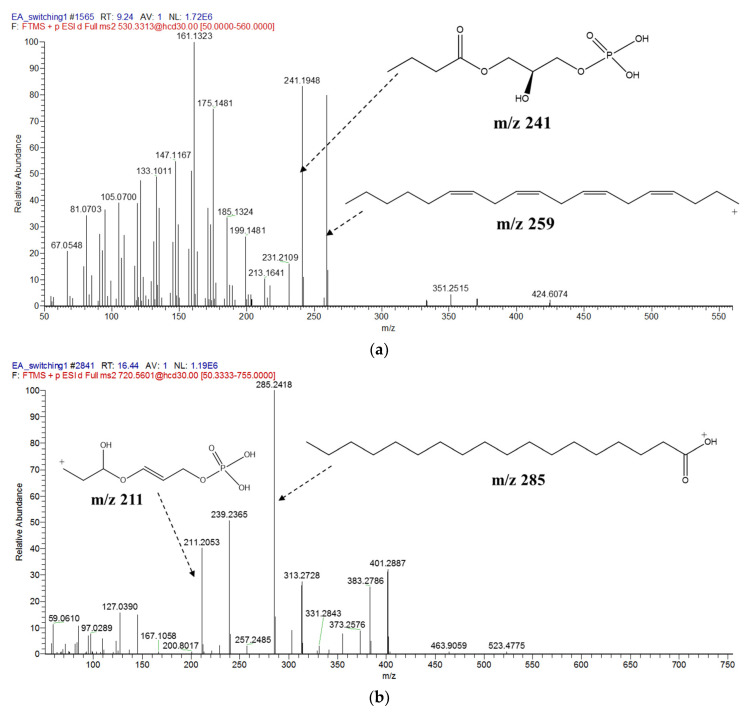
Represent MS/MS spectra showing common fragments shared among (**a**) structures 1–7 and (**b**) structures 8–11 acquired by UPLC–MS/MS in positive mode.

The aim of MN is to compare and group all MS/MS spectra in an individual or series of extracts based on their similarity expressed as cosine score value [39]. In other words, MN is capable of clustering molecules according to their structural characteristics, as its MS/MS spectrum is related to the chemical structure of the fragmented metabolites [79]. Figure 12a presents the MS/MS spectrum of LysoPE(22:4(7Z,10Z,13Z,16Z)/0:0) depicting common fragments found in MS/MS spectra of metabolites number 1 to 7 after they shared several fragments such as *m*/*z* 241 derived from glycerol and phosphate components free from its ethanolamine moiety and another fragment of the hydrocarbon chain-forming *m*/*z* 299 as proposed by Mass Frontier software. Meanwhile, metabolites numbers 8 to 11 share other common fragments, as shown in Figure 12b of the represented MS/MS spectrum of PE(16:0/18:0), showing a fragment of *m*/*z* 211 forming as glycerol and phosphate backbone with hydrogen rearrangement at carbonyl carbon and *m*/*z* 285 established as cleavage of saturated hydrocarbon chain at one of its glycerol ester oxygens. The MS/MS spectrum for all metabolites are provided as Supplementary Files (Figure S4).

### 2.6. Correlation between NMR and UHPLC–MS/MS Data

Advances in analytical methods of high-resolution nuclear magnetic resonance (NMR) spectroscopy and mass spectrometry (MS), with the incorporation of chemometric tools, have driven the field of metabolomics promptly for the high reproducibility of the former and high sensitivity and selectivity of the latter [80,81]. This analytical strategy in metabolomics had been applied in fields like toxicology, drug discovery, early disease detection, and food and nutrition sciences in recent years [82,83,84].

The combination of more than one analytical platform minimizes the shortcomings of using NMR or MS alone [85]. Hyphenation of NMR and MS will permit the exhibition of a diverse range of metabolites [86,87], albeit with a few duplicates, as both eventually offer a comprehensive identification with improved reliability [87,88,89,90]. Furthermore, this approach helps in optimizing the detection of unknown analytes by merging unique NMR information (chemical shifts, coupling constants) with that of MS (exact mass, molecular fragments) [91,92]. A quick approach in biological samples analysis can be achieved using NMR spectroscopy [93], as it can produce fingerprints for samples screening and classification [94]. Further profiling of the significant group of metabolites can then be done using MS.

In the current study, 21 metabolites, comprising amino acids, carbohydrates, fatty acids, and carotenoids, were successfully determined using NMR spectroscopy. Metabolites with a VIP value of more than 1 (Figure 4b) were considered significantly responsible for the separation of the different extracts. The heat map (Figure 5) demonstrates the highest intensity of fatty acids and carotenoids in EtOAc extract, while most of the amino acids were detected in the Aq extract. These results prove the superiority of NMR in identifying the different classes of compounds in fingerprinting analysis.

Low sensitivity is an inherent weakness of NMR spectroscopy, and to make up for this, the EtOAc extract was subjected to MS analysis, a more sensitive analytical tool with a detection level ranging from picomole to femtomole, to further profile the secondary metabolites [95,96]. This high sensitivity, however, may lead to a complicated forest of signals, and MS is also not a universal technique that can detect a wide range of metabolite classes in a single run; to do so, different chromatographic techniques are required [56]. The present study putatively identified thirty-two secondary metabolites by MS, which include carotenoids, chlorophylls, fatty acids, glycerophospholipids, and sphingolipids. The presence of 10 of the identified metabolites, namely palmitic acid, oleic acid, *α*-linolenic acid, arachidic acid, cholesterol, DHA, DPA, fucoxanthin, astaxanthin, and pheophytin a, was verified in the EtOAc extract based on the comparison of the MS data with the NMR. Other carotenoids, such as halocynthiaxanthin, diatoxanthin, and echinenone, as well as chlorophylls, such as pheophorbide a and chlorophyll a, were discovered only by MS technique. Apart from that, MS/MS-based molecular networking approach, which had never been done on *I. galbana,* has succeeded in the discovery of more glycerophospholipids which were classified as phosphatidylethanolamine (PE).

Both NMR and MS analytical platforms had been applied in many studies on algae, such as in the structural elucidation of carotenoids stereoisomers from *Chlorococcum humicola*, a green freshwater algae [97]. In this study, the structure of six closely related carotenoids was successfully characterized and quantified from a mixture. In another study, NMR and GC-MS techniques in combination were used to investigate the quality of lipids and corresponding FAMEs composition in *Chlorella vulgaris*, *Spirulina platensis* and *Tetraselmis affchuii* after treatment in different media composition, to explore their biodiesel potential [98].

## 3. Materials and Methods

### 3.1. Microalgae Culture and Harvest

Isolated *Isochrysis galbana* species (UPMC-A0009) was obtained from the Microalgal Production Laboratory, Aquatic Animal Health Unit, Faculty of Veterinary Medicine, Universiti Putra Malaysia in April, 2017. The stock culture, grown in Conway medium prepared in filtered (5 μm) and sterilized seawater [99], was maintained at 23 °C in an environmental chamber (Sanyo, Osaka, Japan) under 12–12 artificial light–dark cycle (light intensity 150 µmol/m^2^/s). The cultivation of *I. galbana* was initiated from a 25 mL aliquot of the stock culture in Erlenmeyer flasks (5 L) and was gradually scaled up to 90 L under continuous aeration in an annular photobioreactor of 120 L capacity. Biomass weight and cell count were used to monitor the microalgae growth. The former value was determined by dividing the dry weight of the filtered biomass with the filtrate volume [100]. Cell counting was performed daily, on a well-mixed sample, using a Neubauer hemocytometer (Assistant, Germany). The microalgae were harvested at the late-exponential growth phase (15 days), by centrifuging them at 10,000× *g*-force following Aguilera-Sáez et al. (2019) [29] with some modifications. The harvested biomass was then freeze-dried (Scanvac, Lynge, Denmark) and stored at −80 °C before use.

### 3.2. Preparation of Solvent Extracts

Extracts of the dried biomass were prepared in hexane (Hex), ethyl acetate (EtOAc), absolute ethanol (EtOH), EtOH:water 1:1 (AqE), and 100% water (Aq), in 6 replicates for each solvent, giving a total of thirty extracts of five different solvent polarities. Briefly, 200 mg of the dried biomass was mixed with 30 mL solvent in a 50 mL Schott test tube and sonicated in an ultrasonic bath sonicator (Kudos, Shanghai, China) for 30 min, taking care to keep the bath temperature maintained below 35 °C to avoid any unwanted decomposition or degradation of the compounds. The extract was filtered, and the residue was extracted again with fresh solvent. The extract filtrates were pooled, rotary-evaporated at 40 °C, freeze-dried, and stored under −20 °C prior to further analysis. The yield of each solvent extract was obtained from the average value of 6 sample replicates extracted in their respective solvents and freeze-dried.

### 3.3. Spectroscopic Measurements

Spectroscopic measurements of all extracts were separately prepared for ^1^H NMR spectroscopy and ultra-high-performance liquid chromatography-mass spectrometry (UHPLCMS/MS).

#### 3.3.1. ^1^H NMR Analysis

Ten milligrams of each extract was vortex-mixed in microcentrifuge tubes with 700 µL of CD_3_OD:CDCl_3_ at a ratio of 8:2, with 0.05% trimethylsilylpropanoic acid (TSP) as an internal reference standard. The mixture was sonicated for 15 min at room temperature and centrifuged at 18,900× *g*-force for 10 min. About 600 µL of the clear supernatant was transferred to a 5 mm NMR tube for data acquisition [101]. ^1^H-NMR spectra were analysed using 500 MHz Varian Unity INOVA NMR spectrometer (Varian Inc., Palo Alto, CA, USA) functioning at a frequency of 499.91 MHz and maintained at 26 °C. For data acquisition, a single-pulse proton experiment with PRESAT was used with 21.0 µs pulse width, 2-s relaxation delay, 3.53 total acquisition time for 64 scans. Two-dimensional J-resolved experiment (JRES) was used to help clarify the spectral assignment. The time for the J-resolved spectrum acquisition was 50 min and 18 s, with 8 scans per 256 increments for the axis of the spin-spin coupling constant and spectral widths of 66 Hz, and 8 K data points for the chemical shift axis with spectral widths of 8012.8 Hz. The relaxation delay was set at 1.0 s. Heteronuclear single quantum coherence (HSQC) spectra were obtained using 16 scans, 1 K data points, and 256 increments at the spectral width of 13 ppm and 220 ppm for the proton and carbon dimensions, respectively. The relaxation delay was 1.0 s, giving an achievement time of 6 h, 9 min, and 9 s. The 2D NMR spectral processing for structural elucidation was carried out using MestRenova software (version 6.02-5475, Mestrelab Research, Santiago de Compostella, Spain).

#### 3.3.2. LCMS/MS Analysis

Samples were prepared at the concentration of 2 mg/mL in LCMS-grade methanol and ultrasonicated for 10 min before being filtered through a nylon membrane (0.22 mm) into a 2 mL screw-capped sample vial to remove any precipitation. LCMS analysis was performed on a Thermo Scientific^TM^ Q Exactive^TM^ Hybrid Quadrupole-Orbitrap mass spectrometer coupled to a Dionex Ultimate 3000 UHPLC system (Thermo Fisher Scientific Inc., Waltham, MA, USA), which is fitted with Acquity UPLC BEH C18 column (1.7 µm × 2.1 mm × 100 mm) (Waters, Milford, MA, USA). The mobile phase used was 0.1% formic acid in deionized water (solvent A) and 0.1% formic acid in LCMS-grade acetonitrile (solvent B). The injection volume was 5 µL, analysis time was set to 30 min, and the flow rate was 0.25 mL/min. The gradient program commenced with 10% solvent B at 0 min, 20% at 1.00 min, 30% at 2.00 min, 70% at 7.00 min, 80% at 10.00 min, 90% at 12.00 min, and 100% at 13.00–30.00 min. Molecular ion identification was obtained in switching electrospray ionization modes with a full scan range of *m/z* 100–1500 amu. Other MS parameters were set as follows: collision energy of 30 eV, spray voltage of 4.2 kV, capillary temperature at 350 °C, sheath gas flow rate of 50, and auxiliary nitrogen (99% pure) gas flow rate of 10. The mass resolution was set to 70,000 full widths at half maximum (FWHM). The UV detectors were set to 254, 280, 400, and 440 nm, while the PDA detector was set to 190–600 nm. Metabolite assignments were done using the retention time, UV-vis spectra, and MS data (accurate mass, negative and positive ion modes) from the compounds analysed using Thermo Xcalibur 2.0 (Thermo Fisher Scientific Inc., Waltham, MA, USA) and their comparison was performed using literature data and standard online databases (freely available), such as Metabolomics Workbench, Human Metabolome Database (HMDB), PubChem, MassBank, and Metlin. The fragmentation for all compounds discussed herein was based on the pattern derived from HighChem Mass Frontier 3.0 (Thermo Fisher Scientific Inc., Waltham, MA, USA).

### 3.4. Data Processing and Multivariate Data Analysis

Phasing and baseline corrections of NMR spectra were carried out using Chenomx software (version 5.1, Edmonton, AB, Canada). All NMR spectra were phase-adjusted and baseline-corrected automatically and referenced to the internal standard (TSP) at 0.00 ppm. The ^1^H NMR spectrum of each sample was processed and bucketed (bin width of 0.04 ppm) from the spectral region of 0.50 to 10.00 ppm. The peaks for residual water (4.80–4.90 ppm), methanol (3.30–3.32 ppm), and chloroform (7.78–7.79 ppm) were excluded from the spectral data to retain the signals from endogenous metabolites. A total of 243 integrated regions were obtained for each spectrum. The generated dataset was converted to ASCII files and imported to SIMCA-P 13.0 software (Umetrics, Umeå, Sweden) for multivariate data analysis and visualization of results. The dataset was Pareto-scaled, and correlations among the samples were then established by partial least square-discriminant analysis (PLS-DA). A score plot was constructed to visualize the separation between groups, while a loading plot was used to identify metabolites that contributed to the groups’ separation. The model was validated using the default seven-fold internal cross-validation based on the goodness-of-fit (R2X) and goodness-of-prediction (Q2) values together with the 100-permutation test. Hierarchical cluster analysis (HCA) was performed using MetaboAnalyst 3.0 (http://www.metaboanalyst.ca, accessed on 21 Novermber 2019), a public web-based platform for comprehensive analysis of metabolomics data.

### 3.5. Molecular Networking

The molecular networks based on MS/MS data were generated using the online workflow Global Natural Products Social Molecular Networking (GNPS) platform (http://gnps.ucsd.edu, accessed on 28 October 2020) with a registered account. Prior to uploading the data into GNPS, the raw MS data including blank were converted into mzXML format using MSConvert software downloaded from Proteowizard website (http://proteowizard.sourceforge.net/tools.shtml, accessed on 28 October 2020). Then, the converted data files were uploaded to GNPS using FileZilla 3.42.1 software (https://filezilla-project.org/, accessed on 28 October 2020). In the GNPS data analysis workflow, sample and blank data were selected as G1 and G2, respectively, with precursor ion mass tolerance set to 0.02 Da and a fragment ion mass tolerance of 0.02 Da. A network was processed with edges that were filtered to have a cosine score above 0.7 and a minimum 6 matched peaks [71]. Upon processing, the result was downloaded and the network was visualized using ChemViz 1.3 plugin (freely available at http://www.cgl.ucsf.edu/cytoscape/chemViz/, accessed on 28 October 2020) within Cytoscape 3.7.1 software (Institute of Systems Biology, Seattle, WA, USA).

### 3.6. Statistical Analysis

One-way analysis of variance (ANOVA) was performed using GraphPad Prism 6.0 (GraphPad Software, San Diego, CA, USA). Post-hoc analysis was carried out using Tukey’s test, wherein values with *p* ≤ 0.05 were considered to be statistically significant. Values were expressed as mean ± standard deviation (SD).

## 4. Conclusions

To the best of our knowledge, this is the first report describing the comprehensive metabolites profiling of *I. galbana* by NMR and supplemented with LCMS. The extraction of *I. galbana* in five different solvent polarities successfully exhibited the highest concentrations of fatty acids and carotenoids in EtOAc extract and amino acids and carbohydrates in Aq extract, as determined by the NMR fingerprints. Hence, both solvents are preferable for the extraction of these microalgae to obtain a wide range of compounds. The identification of important metabolites in EtOAc extract was further confirmed using UHPLC–MS/MS analysis, from which another 32 metabolites comprising fatty acids, carotenoids, glycerophospholipids, and sphingolipids were identified, with 10 of them present in both of the analyses. Further identification using MN platform on MS/MS data has discovered more glycerophospholipids that are classified as PE. The NMR fingerprinting together with MS profiling managed to fruitfully characterize a large set of metabolites with a wide range of classes while improving the accuracy of the ones identified. The understanding of metabolites content in *I. galbana* brought about in this study can be utilized for the preparation of a standardized feed for selected aquaculture assays.

## Figures and Tables

**Table 1:** Yield for each solvent extract.

Solvent	Aq	AqE	EtOH	EtOAc	Hex
Yield (g)	0.068 ± 0.003 ^d^	0.042 ± 0.001 ^ab^	0.057 ± 0.002 ^c^	0.047 ± 0.001 ^b^	0.038 ± 0.002 ^a^

Data are expressed as mean ± SD. Values with the different superscript letters are significantly different as determined by Turkey test (*p* < 0.05).

**Table 2:** Characteristic ^1^H NMR signals of metabolites identified in various solvent extracts of *I. galbana* (s—singlet; d—doublet; t—triplet; dd—doublet of doublets; m—multiplet; J—coupling constant).

No.	Putative Metabolite	δH (ppm), Multiplicity, *J* (Hz)	HSQC (^1^H-^13^C)	Hex	EtOAc	EtOH	AqE	Aq	Reference
**Amino acids**									
1.	Valine	0.99 (d, 6.2)	25.1	-	-	+	+	+	[29]
		1.05 (d, 2.8)	21.1						
2.	Isoleucine	1.01 (d, 3.3)	19.5	-	-	+	+	+	[29]
		0.96 (t, 7.6)	-						
3.	Leucine	0.97 (d, 5.0)	23.8	+	+	+	+	+	[29]
4.	Alanine	3.74 (m)	72.8	-	-	+	+	+	[44]
		1.46 (d, 7.2)	19.1						
5.	Threonine	3.64 (dd, 5.7, 10.9)	66.5	-	-	-	-	+	[44]
		1.37 (d, 6.9)	-						
**Carbohydrates/Others**									
6.	Sucrose	5.37 (d, 4.1)	-	-	-	+	+	+	[45]
		4.22 (d, 2.5)	76.5						
		3.83 (m)	-						
		3.68 (s)	-						
		3.44 (t, 6.7)	74.5						
7.	Glucose	3.78 (m)	72.8	-	-	+	+	+	[45]
		3.52 (dd, 4.6, 8.4)	66.5						
8.	Choline	3.22 (s)	57.0	-	-	+	+	+	[29]
9.	Dimethylsulphonio-propionate (DMSP)	3.44 (t, 6.7)	43.9	-	-	+	+	+	[29]
		2.92 (s)	28.3						
		2.70 (t, 6.7)	33.4						
10.	d-1,4/2,5-cyclohexa-netetrol	3.72 (m)	73.2	-	-	+	+	+	[29]
		1.83 (m)	37.3						
**Saturated fatty acids**									
11.	Palmitic acid	2.35 (t, 7.6)		+	+	+	+	-	[45]
		1.61 (m)	27.9						
		1.33 (m)	33.2						
		0.88 (t, 6.9)	16.4						
12.	Arachidic acid	2.35 (t, 7.6)		+	+	+	+	-	[45]
		1.60 (m)	27.9						
		1.40 (m)	18.2						
		1.29 (m)	32.2						
		0.88 (t, 6.9)	16.4						
**Unsaturated fatty acids**									
13.	Oleic acid	5.34 (m)	130.3	+	+	+	+	-	[45]
		2.25 (t, 7.5)	37.4						
		2.02 (m)	23.3						
		1.27 (m)	33.2						
		0.87 (t, 6.9)	16.4						
14.	α-linoleic acid	5.34 (m)	-	+	+	+	+	-	[45]
		5.32 (m)	-						
		2.80 (m)	29.1						
		2.25 (t, 7.5)	37.4						
		2.02 (m)	-						
		1.27 (m)	-						
		0.96 (t, 7.6)	16.6						
15.	Docosahexaenoic fatty acid chain (DHA)	5.30–5.38 (m)	130.3	+	+	+	+	-	HMDB
		2.38 (m)	36.8						
		2.06 (m)	29.7						
		0.96 (t, 7.6)	16.6						
16.	Docosapentaenoic fatty acid chain (DPA)	5.30–5.38 (m)	131.8	+	+	+	+	-	HMDB
		2.33 (m)	36.9						
		1.38 (m)	31.3						
		1.30 (m)	33.2						
		0.87 (t, 6.9)	32.0						
17.	Cholesterol	5.28 (dd, 8.3, 17.6)	-	+	+	+	+	-	HMDB
		2.31 (dd, 2.8, 6.2)	-						
		1.61 (m)	-						
		0.82 (m)	22.2						
		0.77 (d, 6.6)	25.0						
		0.65 (m)	-						
**Carotenoids**									
18.	Fucoxanthin	6.77 (dd, 4.2, 4.2)	-	-	+	+	-	-	[29], HMDB
		6.41 (dd, 11.1, 22.2)	-						
		2.59 (d, 18.5)	-						
		2.12 (s)	-						
		2.09 (s)	-						
		1.97 (s)	-						
		1.58 (dd, 7.2, 13.7)	-						
		1.37 (dd, 5.7, 9.7)	31.3						
		1.19 (s)	23.1						
		1.08 (s)	34.7						
		1.01 (s)	26.9						
19.	Astaxanthin	6.77 (m)	-	+	+	+	-	-	[45], HMDB
		6.61 (d, 3.8)	-						
		6.63 (d, 3.2)	-						
		4.15 (dd)	65.2						
		2.12 (s)	-						
		1.91 (s)	13.8						
		1.19 (s)	23.1						
**Chlorophylls**									
20.	Pheophytin a	9.21 (s)	107.2	+	+	+	-	-	[46], PubChem
		9.00 (s)	99.9						
		8.60 (s)	96.2						
**Alkenone**									
21.	Polyunsaturated long-chain alkenones (PULCAs)	2.12 (s)	-	+	+	+	-	-	[46], PubChem
		1.95 (m)	35.4						

Positive (+) and negative (-) signs denote present and absent, respectively; Ref. = Reference.

**Table 3:** Putative metabolites identified in EtOAc extract of *I. galbana* by LCMS/MS in positive and negative mode; n.d. = not detected. (Exp. = experimental; Theo = theoretical).

Peak	t_R_ (min)	Putative Metabolite	Exp. Mass (M + H)	Exp. Mass (M − H)	Theo. Mass (M +/− H)	Mass Error (ppm)	MS Fragments (ESI^+^)	UV (nm)	MAIN CLASS
1.	7.00	Arachidic acid		311.1688	311.3028	−430	293, 267, 249, 223	n.d	Fatty acid
2.	10.11	Astaxanthin	597.3928		597.3938	−1.67	597, 579, 279, 215, 109	438	Carotenoid
3.	10.18	Halocynthiaxanthin	599.4077		599.4095	−3.00	389, 233, 147, 109	448	Carotenoid
4.	10.71	3,6,9,12,15-Octadecapentaenoic acid		273.1861	273.1860	0.36	228, 182, 133, 59	n.d	Fatty acid
5.	11.40	Chlorophyll c2	609.1970		609.1983	−2.13	591, 549, 532	450	Chlorophyll
6.	11.21	Stearidonic acid		275.2015	275.2017	−0.72	83, 71, 59	n.d	Fatty acid
7.	11.52	Chlorophyll c1	611.2125		611.2139	−2.25	593, 551, 534	446	Chlorophyll
8.	11.90	Cholesterol	387.1795		387.3548	−452	387, 362, 207	n.d	Sterol
9.	12.05	Eicosapentaenoic acid (EPA)		301.2173	301.2173	0	187, 166, 148	n.d	Fatty acid
10.	12.07	Fucoxanthin	659.4281		659.4306	−3.79	581, 411, 355, 199, 109	448	Carotenoid
11.	12.08	*α*-Linolenic acid		277.2172	277.2173	−0.36	194, 92, 87, 59	n.d	Fatty acid
12.	12.49	Docosahexaenoic acid (DHA)		327.2328	327.2330	−0.61	213, 172, 135, 59	n.d	Fatty acid
13.	13.10	Pheophorbide a	593.2742		593.2758	−2.69	593, 533, 506, 459	408, 536	Chlorophyll
14.	13.29	Docosapentaenoic acid (DPA)		329.2487	329.2486	−0.30	250, 226, 85, 59	n.d	Fatty acid
15.	13.37	(3*S*,4*R*,3′*R*)-4-Hydroxyalloxanthin	581.3976		581.3989	−2.23	563, 411, 251	438	Carotenoid
16.	13.60	Phoenicoxanthin	581.3964		581.3989	−4.30	411, 429	452	Carotenoid
17.	13.82	Palmitic acid		255.2329	255.2330	−0.39	246, 94, 81, 76	n.d	Fatty acid
18.	13.91	Oleic Acid		281.2486	281.2486	0	101,98, 87, 64	n.d	Fatty acid
19.	15.26	Hexacosanedioic acid	427.3771		427.3782	−2.57	203, 139, 121, 71	n.d	Fatty acid
20.	15.83	Diatoxanthin	567.4182		567.4197	−2.64	255, 211, 119, 109	460	Carotenoid
21.	15.92	Canthaxanthin	565.4023		565.4040	−3.00	447, 255, 119	456	Carotenoid
22.	16.45	Echinenone	551.4232		551.4247	−2.72	551, 502, 458, 447	458	Carotenoid
23.	17.56	PI(16:0/22:4(7Z,10Z,13Z,16Z))	887.5657		887.5644	1.35	871, 609, 591	n.d	Glycerophospholipids
24.	17.90	PC(15:1(9Z)/22:6(4Z,7Z,10Z,13Z,16Z,19Z))	790.5438		790.5381	7.21	628, 610, 356	n.d	Glycerophospholipids
25.	18.68	PI-Cer(d14:0/31:0)	936.7314		936.7264	5.33	919, 643, 591	n.d	Sphingolipids
26.	19.82	PS(O-14:0/26:0)	834.6580		834.6582	−0.23	698, 589, 543	n.d	Glycerophospholipids
27.	20.51	Chlorophyll a	893.5512		893.5426	9.62	893, 615, 539	408, 536	Chlorophyll
28.	21.06	Pheophytin a	871.5711		871.5732	−2.41	871, 593, 533	408, 536	Chlorophyll
29.	21.59	PI-Cer(d14:0/28:0)	930.6123		930.6196	−7.84	631, 603, 506		Sphingolipids
30.	22.80	NAPE(18:1(9Z)/16:1(9Z)/18:0)	982.7805		982.7834	−2.95	921, 828, 636		Glycerophospholipids
31.	24.63	PI-Cer(t18:0/16:0(2OH))	814.5496		814.5440	6.87	797, 569, 543		Sphingolipids
32.	28.47	TG(12:0/16:0/22:5(7Z,10Z,13Z,16Z,19Z))	825.6924		825.6967	−5.21	698, 597, 549		Glycerolipids

**Table 4:** Putative annotation of metabolites from a cluster of glycerophospholipids identified in EtOAc extract of *I. galbana*.

No.	Parent Mass *m*/*z* [M + H]^+^	Mass Error (ppm)	Putative Annotation	Molecular Formula	RT (min)
1.	530.3313	13.58	LysoPE(22:4(7Z,10Z,13Z,16Z)/0:0)	C_27_H_48_NO_7_P	9.24
2.	788.5286	7.74	PE(22:6(4Z,7Z,10Z,13Z,16Z,19Z)/ 18:2(9Z,12Z))	C_45_H_74_NO_8_P	14.38
3.	609.4493	−3.28	DG(18:4(6Z,9Z,12Z,15Z)/18:4 (6Z,9Z,12Z,15Z)/0:0)	C_39_H_60_O_5_	15.00
4.	794.5761	8.43	PE(20:2(11Z,14Z)/20:3(8Z,11Z,14Z))	C_45_H_80_NO_8_P	15.47
5.	840.5593	6.54	PE(22:4(7Z,10Z,13Z,16Z)/22:6 (4Z,7Z,10Z,13Z,16Z,19Z))	C_49_H_78_NO_8_P	14.74
6.	768.5600	8.07	PE(18:0/20:4(5Z,8Z,11Z,14Z))	C_43_H_78_NO_8_P	15.85
7.	740.5287	8.37	PE(14:0/22:4(7Z,10Z,13Z,16Z))	C_41_H_74_NO_8_P	14.93
8.	720.5601	8.74	PE(16:0/18:0)	C_39_H_78_NO_8_P	16.44
9.	746.5757	8.44	PE(18:0/18:1(9Z))	C_41_H_80_NO_8_P	16.55
10.	742.5446	8.75	PE(16:1(9Z)/20:2(11Z,14Z))	C_41_H_76_NO_8_P	15.58
11.	692.529	9.39	PE(16:0/16:0)	C_37_H_74_NO_8_P	15.67

## Supplementary Materials

The following are available online at https://www.mdpi.com/1660-3397/19/3/139/s1, Figure S1: (a) R2 and Q2 values from summary of fit model and (b) the R2 and Q2 intercepts derived from 100 permutation tests for each of the solvent extracts; Figure S2: VIP plot with values more than 1.0 and error bars not crossing baseline; Figure S3: MS/MS spectra of molecular ion detected by LCMS/MS in positive and negative mode; Figure S4: MS/MS spectra of metabolites from a cluster of glycerophospholipids identified in EtOAc extract of *I. galbana* in positive mode; Figure S5: Full molecular network of EtOAc extract of *I. galbana*; Figure S6: Representatives 1D 500 MHz ^1^H NMR individual spectrum.
