# Supplementary material for: Complementary Analytical Platforms of NMR Spectroscopy and LCMS Analysis in the Metabolite Profiling of Isochrysis galbana
Source: Mar Drugs. 2021 Mar 2;19(3):139. doi: 10.3390/md19030139 (PMC7998644; doi:10.3390/md19030139)
Supplement: Supplementary file 1 [file marinedrugs-19-00139-s001.pdf]

Complementary analytical platforms of H NMR spectroscopy and LCMS analysis in the metabolite profiling of *Isochrysis galbana*

Muhammad Safwan Ahamad Bustamam[1], Ahmed Hamza Pantami[2], Awanis Azizan[1], Khozirah Shaari[1,2], Chong Chou Min[3], Faridah Abas[1], Norio Nagao[3], Maulidiani M.[4], Sanjoy Banerjee[1], Fadzil Sulaiman[1] and Intan Safinar Ismail[1,2]*

[1] Laboratory of Natural Products, Institute of Bioscience, Universiti Putra Malaysia, 43400 UPM Serdang, Selangor, Malaysia.

[2] Department of Chemistry, Faculty of Science, Universiti Putra Malaysia, 43400 Serdang, Selangor, Malaysia

[3] Department of Aquaculture, Faculty of Agriculture, Universiti Putra Malaysia, 43400 Serdang, Selangor, Malaysia

[4] Faculty of Science and Marine Environment, Universiti Malaysia Terengganu

Correspondence: Intan Safinar Ismail, Laboratory of Natural Products, Institute of Bioscience, Universiti Putra Malaysia, 43400 Serdang, Selangor

Tel.: +60-3-8947-1490**; Fax:** +60-3-8942-3552

Email address: safinar@upm.edu.my

a)

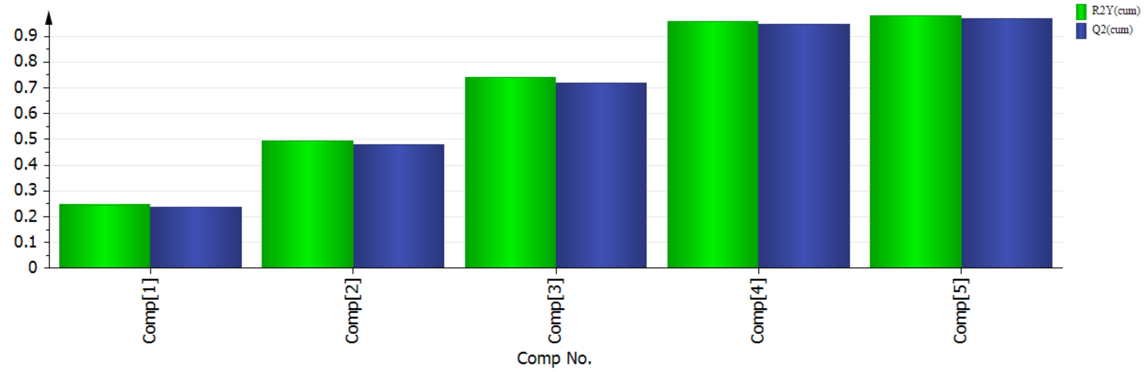

b)

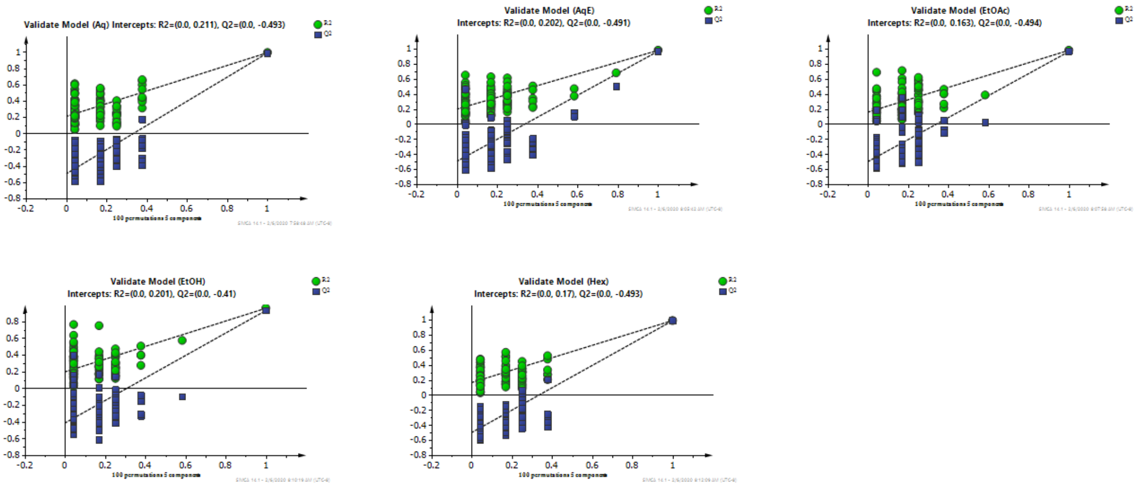

Figure S1 (a) R2 and Q2 values from summary of fit model and (b) the R2- and Q2- intercepts derived from 100 permutation tests for each of the solvent extracts

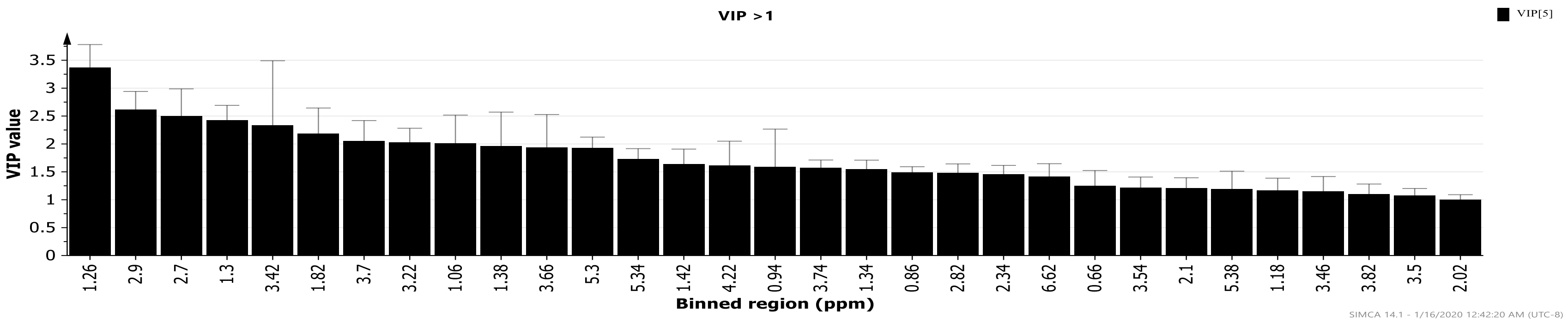

Binned region (ppm)	VIP value	Tentative metabolite
1.26	3.37	Fatty acid derivataties
2.9	2.61	DMSP
2.7	2.49	DMSP
1.3	2.43	Fatty acid derivataties
3.42	2.33	DMSP
1.82	2.18	D-1,4/2,5-cyclohexanetetrol
3.7	2.05	Amino acid derivataties
3.22	2.02	Choline
1.06	2.01	valine/violaxanthin
1.38	1.96	Threonine/DPA/Fucoxanthin
3.66	1.93	Sucrose
5.3	1.92	Fatty acid derivataties
5.34	1.73	Fatty acid derivataties
1.42	1.64	Arachidic acid
4.22	1.61	Sucrose
0.94	1.59	Leucine/α-linoleic acid

Binned region (ppm)	VIP value	Tentative metabolite
3.74	1.57	D-1,4/2,5-cyclohexanetetrol
1.34	1.54	Palmitic acid
0.86	1.48	Palmitic acid
2.82	1.48	α-linoleic acid
2.34	1.45	Fatty acid derivataties
2.94	1.45	DMSP
6.62	1.41	Astaxanthin
0.66	1.25	Cholesterol
3.54	1.21	Glucose
2.1	1.20	Carotenoid derivatives
5.38	1.19	Sucrose/DHA/DPA
1.18	1.16	Carotenoid derivatives
3.46	1.15	DMSP
3.82	1.10	Glucose
3.5	1.08	Amino acid derivatatives
2.02	1.00	Oleic acid

Figure S2 VIP plot with values more than 1.0 and error bars not crossing baseline

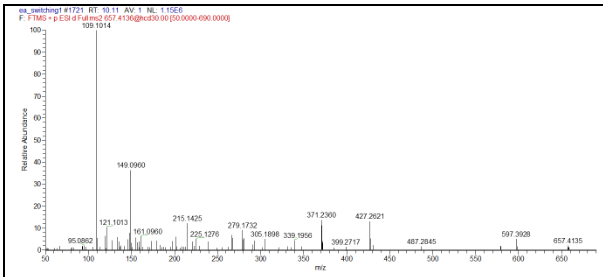

1. m/z 597.3928

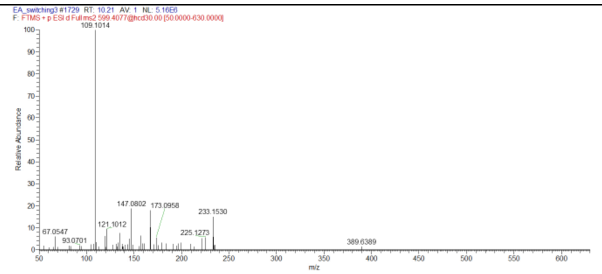

2. m/z 599.4077

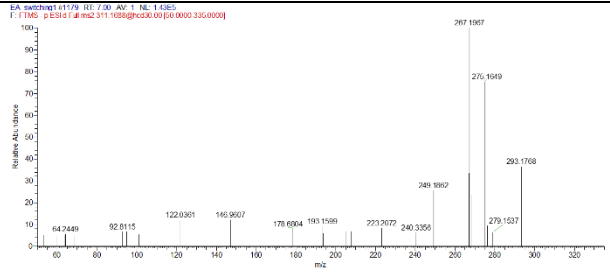

3. m/z 311.1688

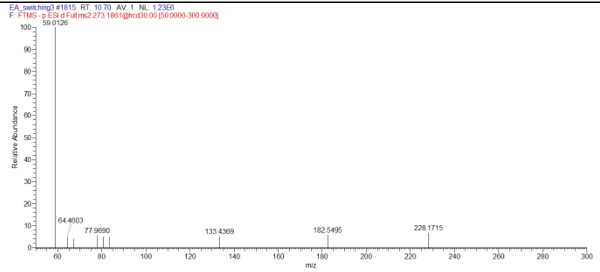

4. m/z 273.1861

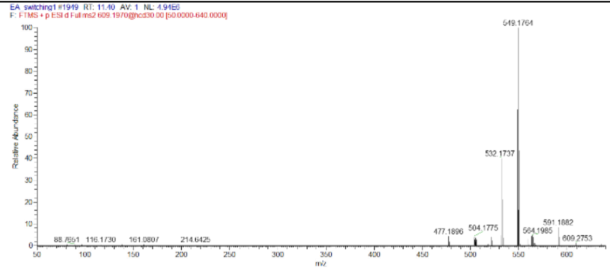

5. m/z 609.1970

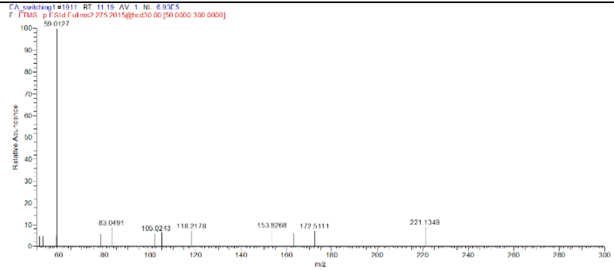

6. m/z 275.2015

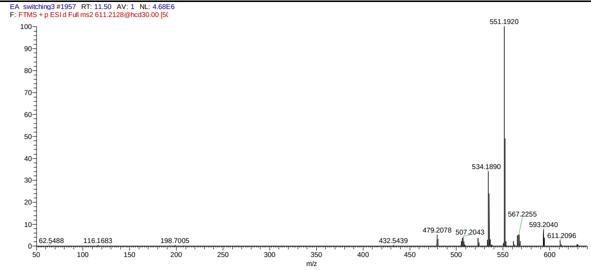

7. m/z 611.2125

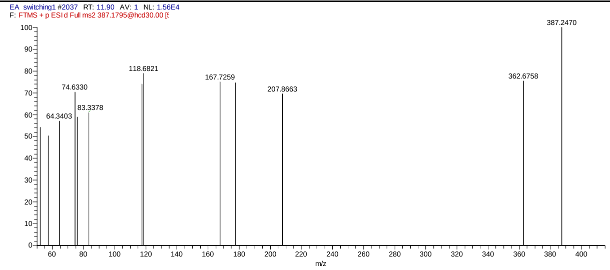

8. m/z 387.1795

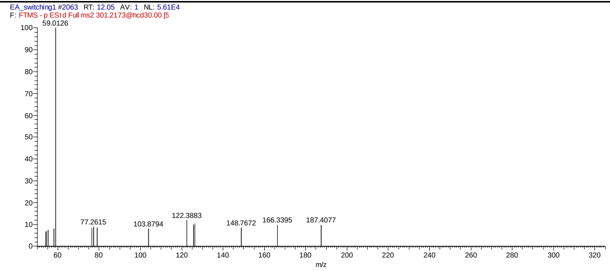

9. m/z 301.2173

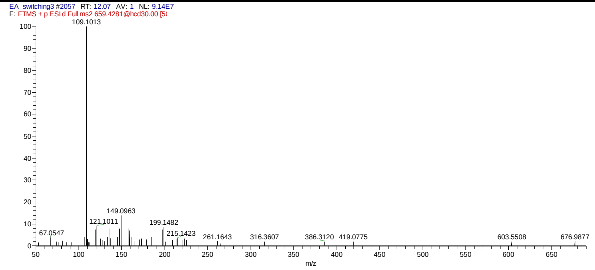

10. m/z 659.4281

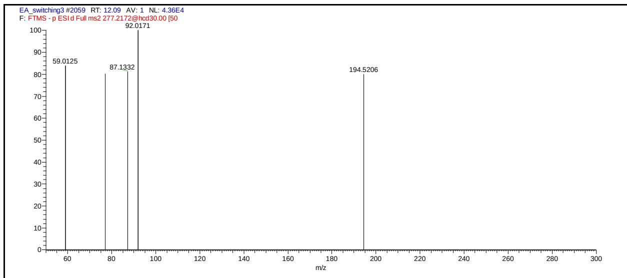

11. m/z 277.2172

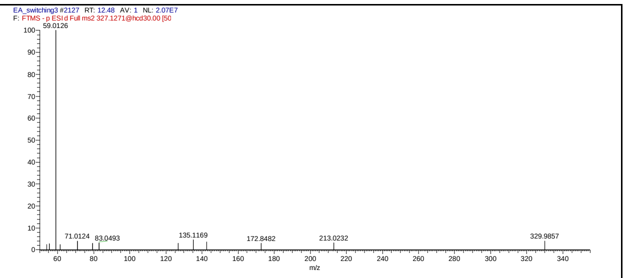

12. m/z 327.2328

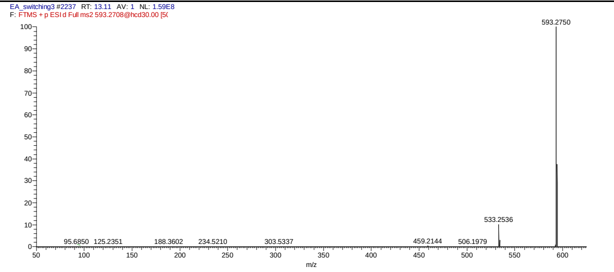

13. m/z 593.2742

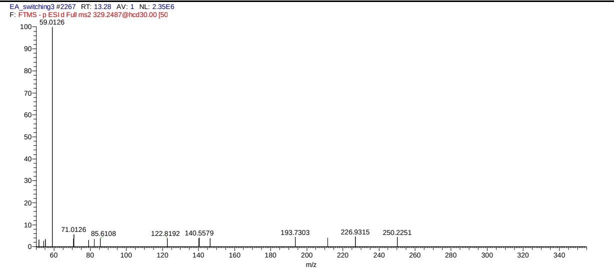

14. m/z 329.2487

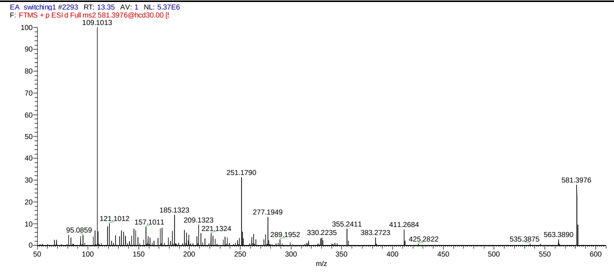

15. m/z 581.3976

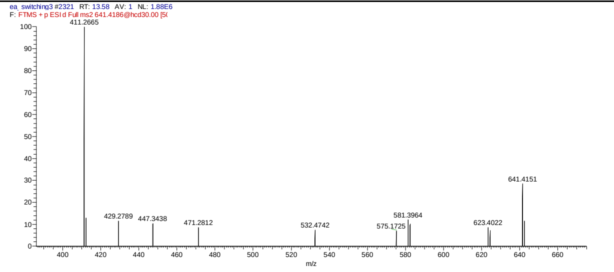

16. m/z 581.3964

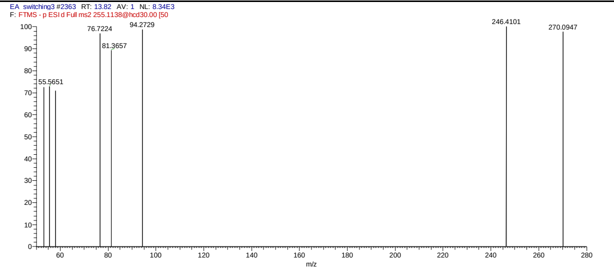

17. m/z 255.2329

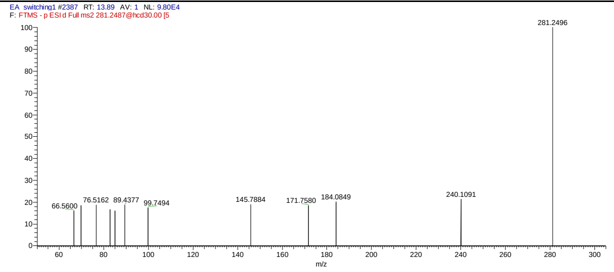

18. m/z 281.2486

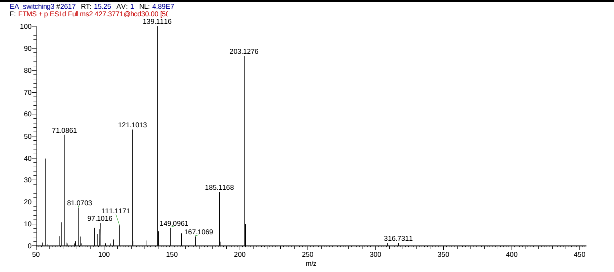

19. m/z 427.3771

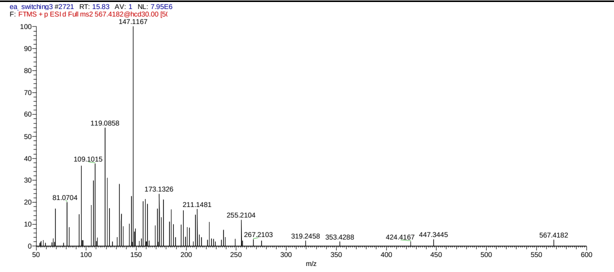

20. m/z 567.4182

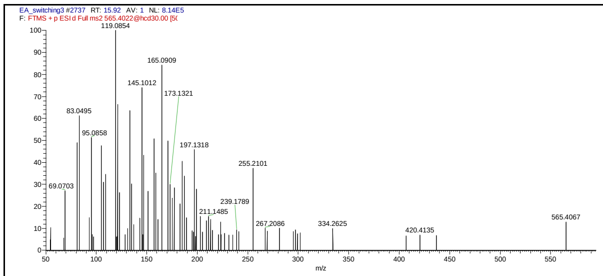

21. m/z 565.4023

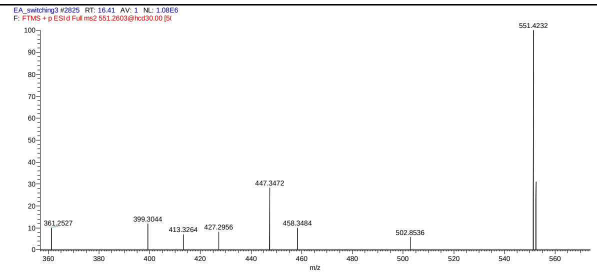

22. m/z 551.4232

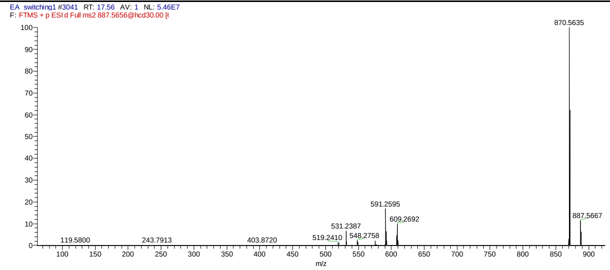

23. m/z 887.5657

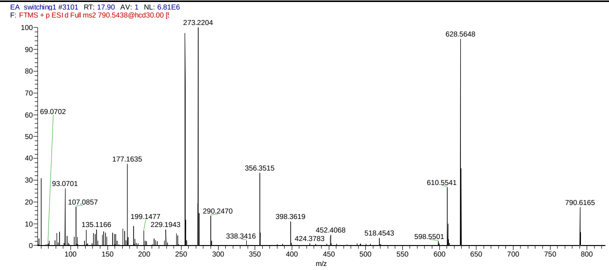

24. m/z 790.5438

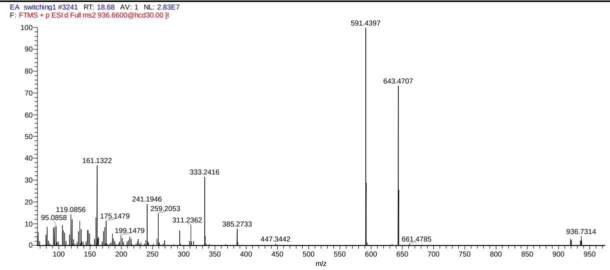

25. m/z 936.7314

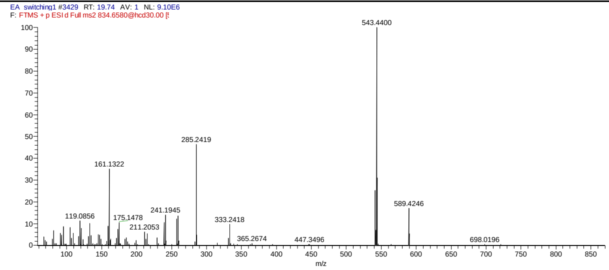

26. m/z 834.6580

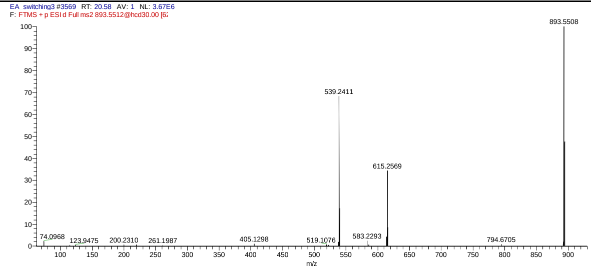

27. m/z 893.5512

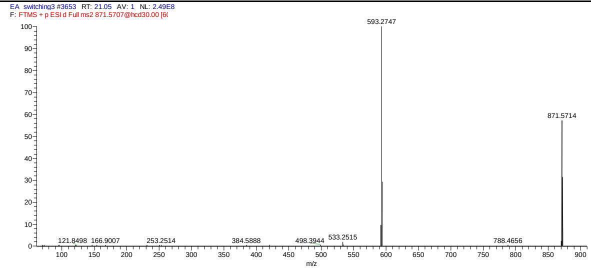

28. m/z 871.5711

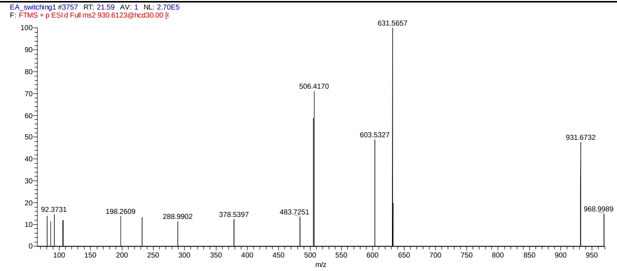

29. m/z 930.6123

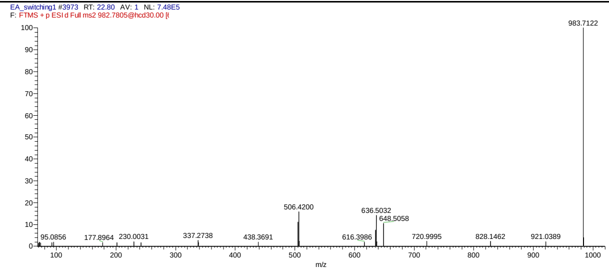

30. m/z 982.7805

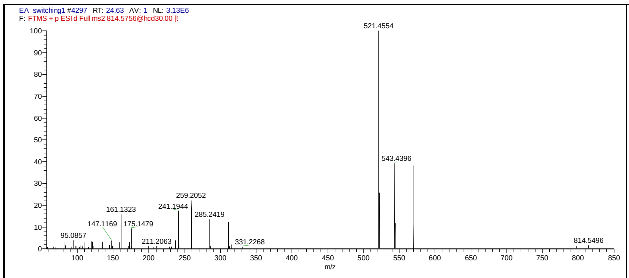

31. m/z 814.5496

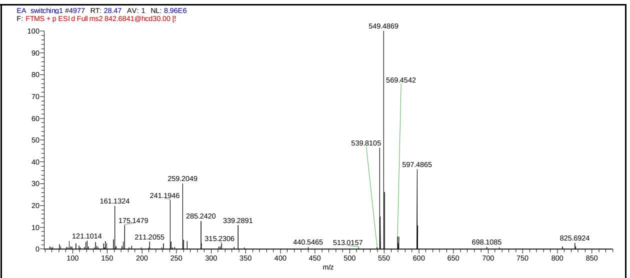

32. m/z 825.6924

Figure S3 MS/MS spectrums of molecular ion detected by LCMS/MS in positive and negative mode

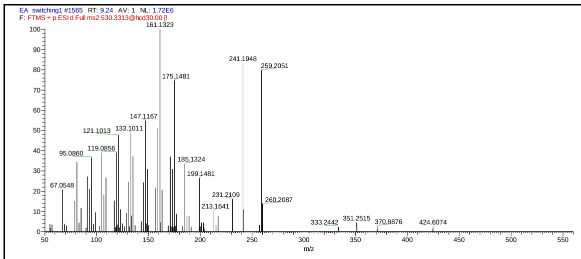

1. m/z 530.3133

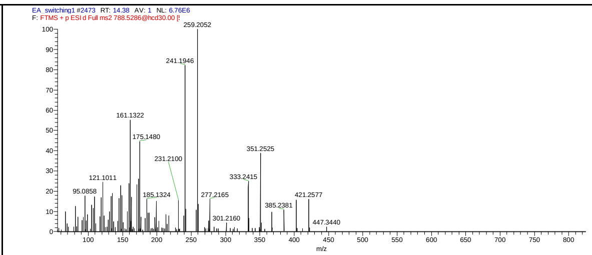

2. m/z 788.5286

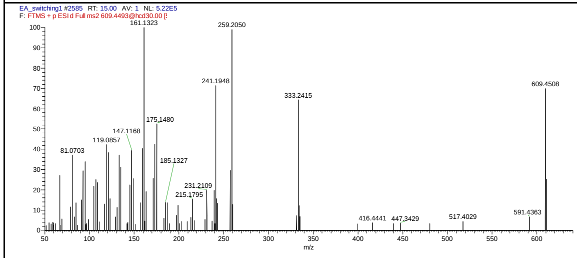

3. m/z 609.4493

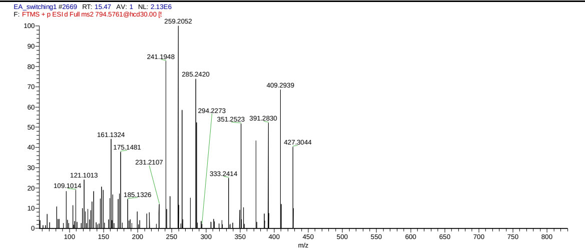

4. m/z 794.5761

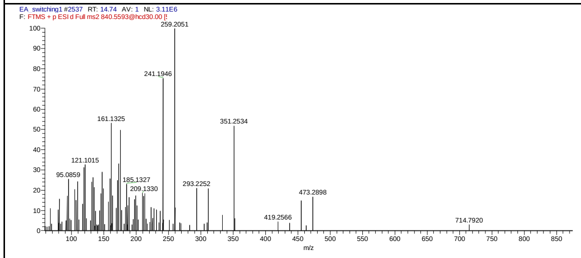

5. m/z 840.5593

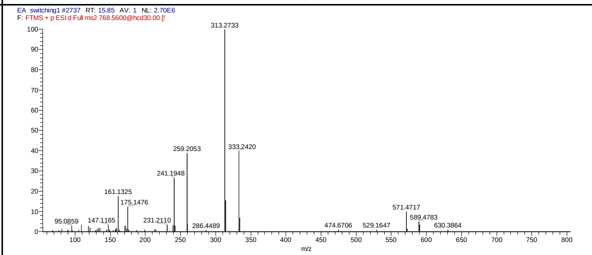

6. m/z 756.00

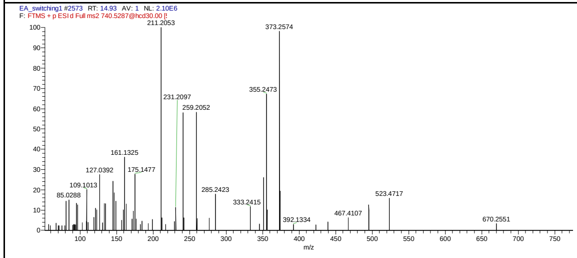

7. m/z 740.5287

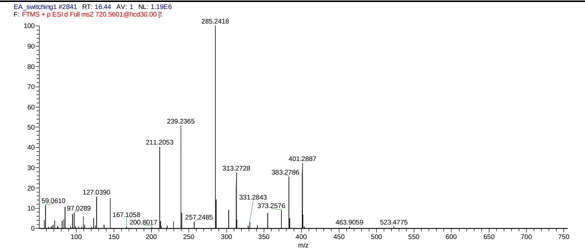

8. m/z 720.5601

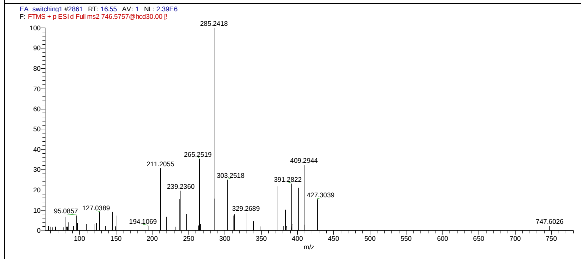

9. m/z 746.5757

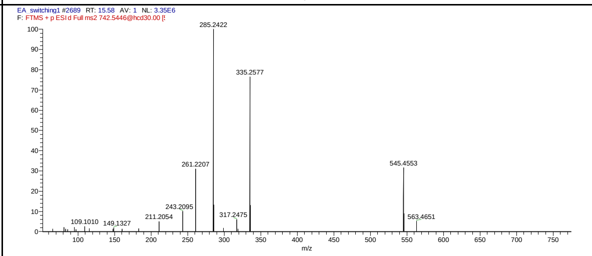

10. m/z 742.5446

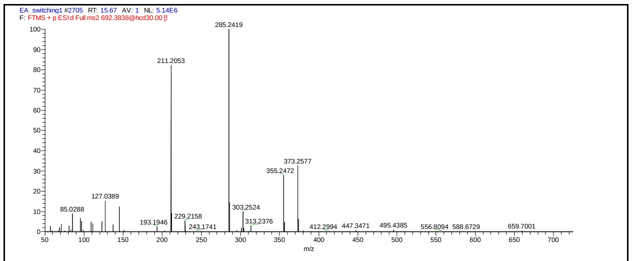

11. m/z 692.3838

Figure S4 MS/MS spectrums of metabolites from cluster of glycerophospholipids identified in EtOAc extract of *I. galbana*. in positive mode

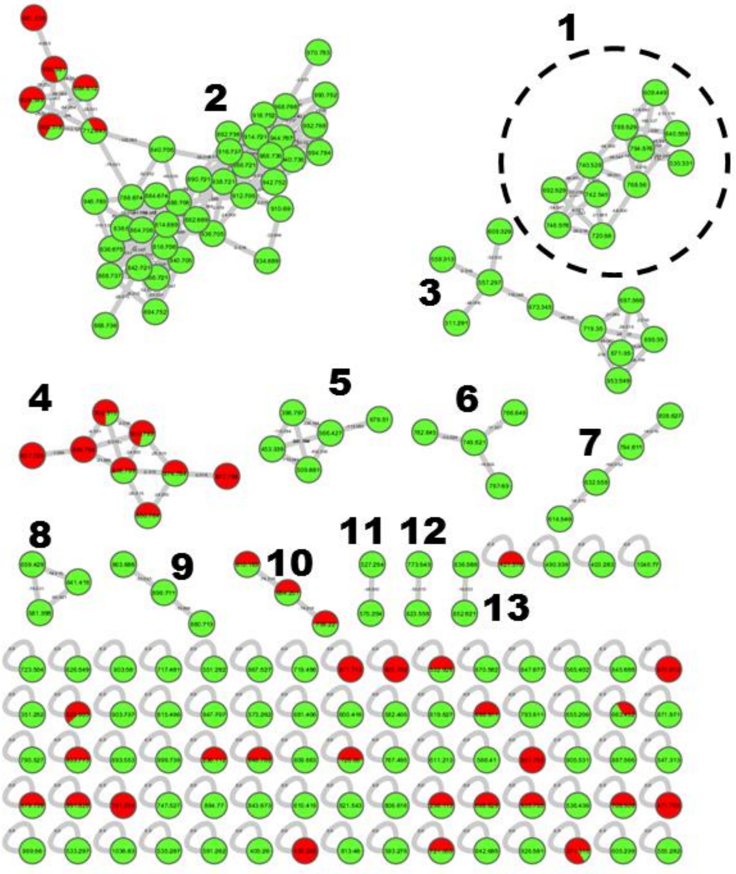

Figure S5 Full molecular network of EtOAc extract of *I. galbana* showing 13 clusters including cluster of glycerophospholipids (cluster no. 1) with 11 new identified putative structures. Green node is EtOAc extract and red node is blank sample.

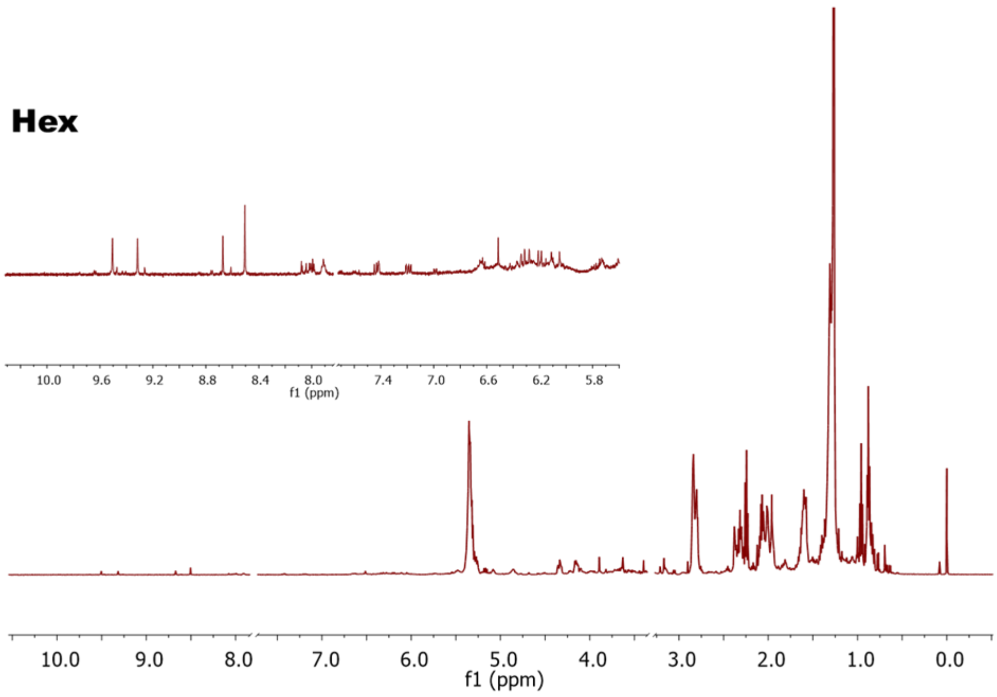

Hex
10.0
9.6
9.2
8.8
8.4
8.0
7.4
7.0
6.6
6.2
5.8
f1 (ppm)
10.0
9.0
8.0
7.0
6.0
5.0
4.0
3.0
2.0
1.0
0.0
f1 (ppm)

(a)

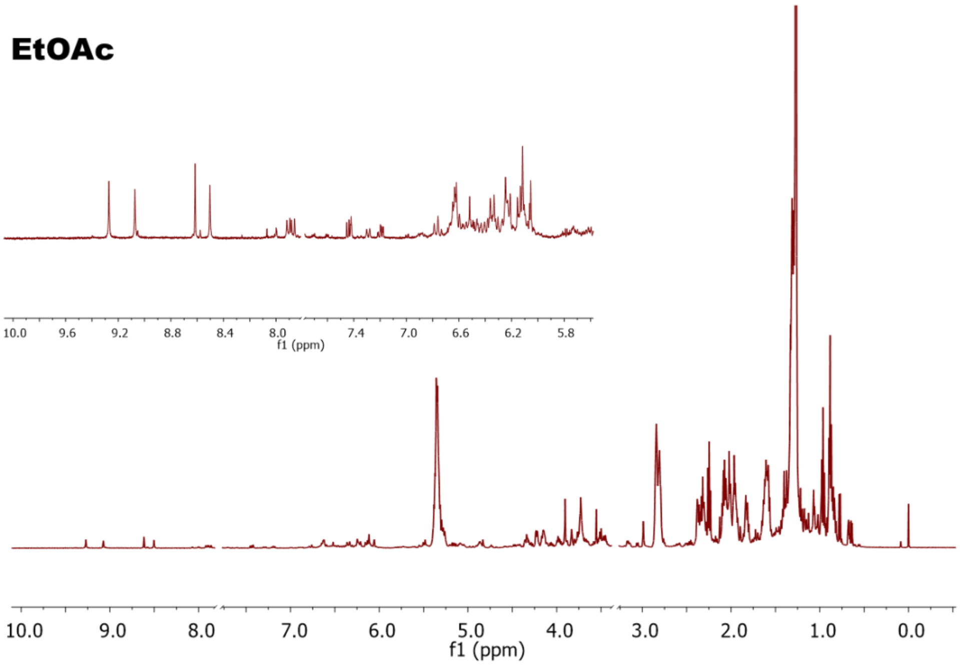

EtOAc
10.0
9.6
9.2
8.8
8.4
8.0
7.4
7.0
6.6
6.2
5.8
f1 (ppm)
10.0
9.0
8.0
7.0
6.0
5.0
4.0
3.0
2.0
1.0
0.0
f1 (ppm)

(b)

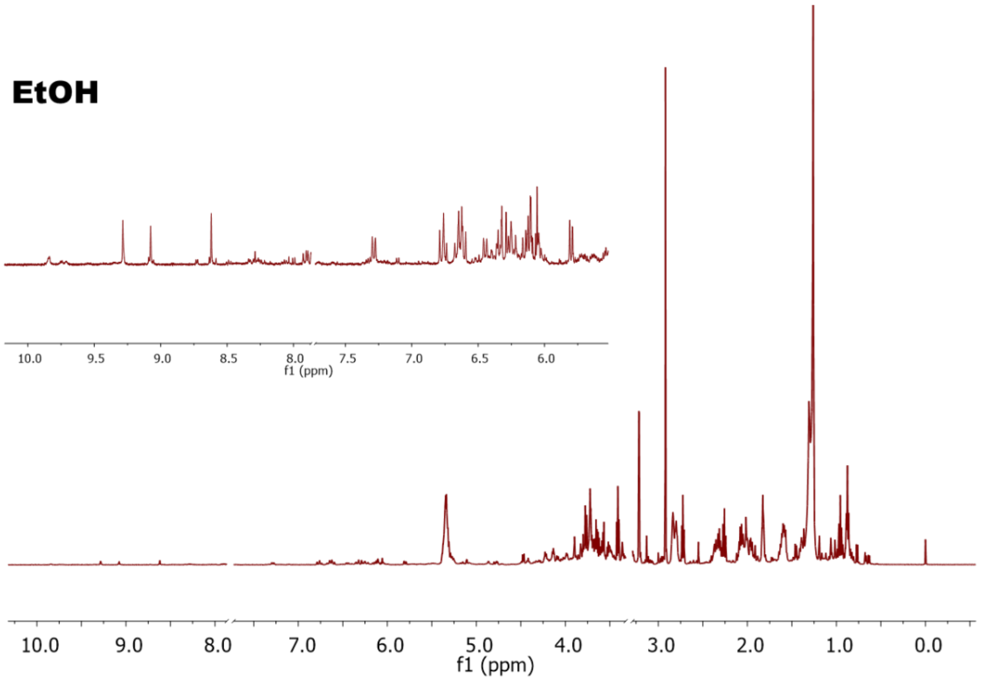
EtOH
10.0 9.5 9.0 8.5 8.0 7.5 7.0 6.5 6.0
f1 (ppm)
10.0 9.0 8.0 7.0 6.0 5.0 4.0 3.0 2.0 1.0 0.0
f1 (ppm)

(c)

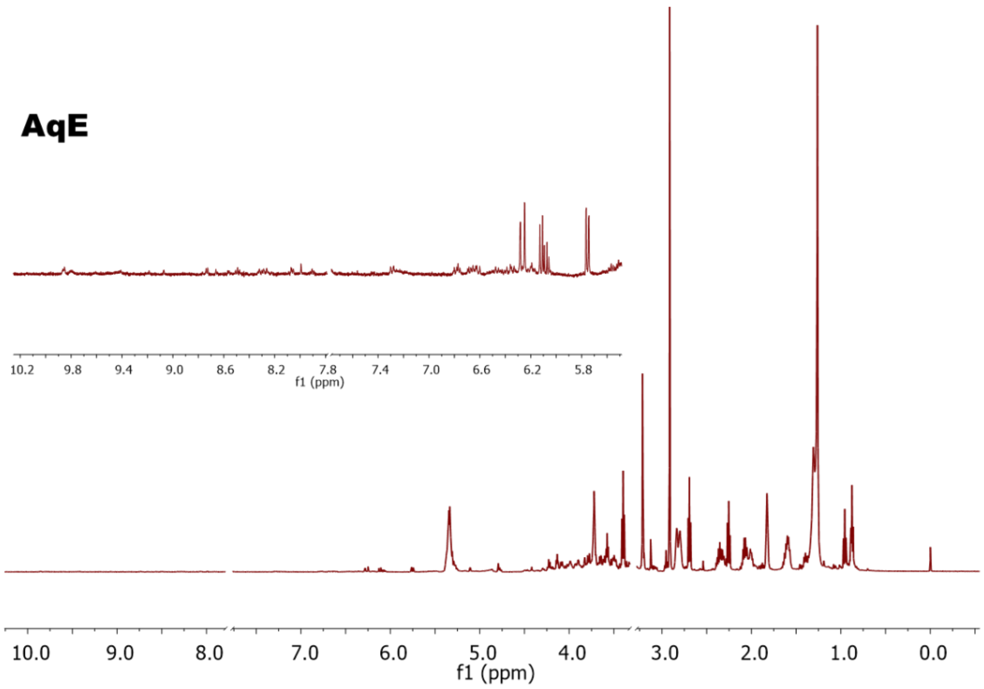
AqE
10.2 9.8 9.4 9.0 8.6 8.2 7.8 7.4 7.0 6.6 6.2 5.8
f1 (ppm)
10.0 9.0 8.0 7.0 6.0 5.0 4.0 3.0 2.0 1.0 0.0
f1 (ppm)

(d)

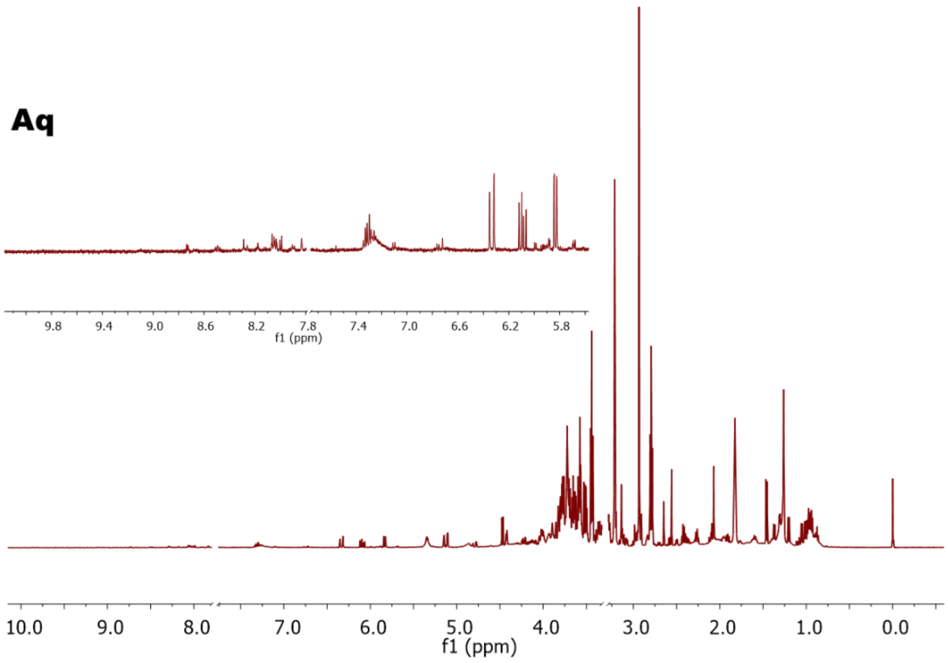

(e)

Figure S6 Representatives 1D 500 MHz ^{1}H NMR individual spectrum of (a) hexane (Hex), (b) ethyl acetate (EtOAc), (c) absolute ethanol (EtOH), (d) 50% ethanol (AqE) and (e) aqueous (Aq) extracts of *I. galbana*.
